# Anti-Tumor Action, Clinical Biochemistry Profile and Phytochemical Constituents of a Pharmacologically Active Fraction of *S*. *crispus* in NMU-Induced Rat Mammary Tumour Model

**DOI:** 10.1371/journal.pone.0126426

**Published:** 2015-05-22

**Authors:** Nik Soriani Yaacob, Hassan Muhammad Yankuzo, Sutha Devaraj, Jimmy Ka Ming Wong, Choon-Sheen Lai

**Affiliations:** 1 Department of Chemical Pathology, School of Medical Sciences, Universiti Sains Malaysia, Kubang Kerian, Kelantan, Malaysia; 2 Centre for Drug Research, Universiti Sains Malaysia, Pulau Pinang, Malaysia; University of Texas Health Science Center, UNITED STATES

## Abstract

Cancer patients seek alternative remedies such as traditional medicinal plants for safe and effective treatment and help overcome the side effects of conventional therapy. Current knowledge indicates that extracts of *Strobilanthes crispus* of the Acanthaceae family exhibit potent anticancer properties *in vitro* and are non-toxic *in vivo*. *S*. *crispus* was also reported to be protective against chemical hepatocarcinogenesis. We previously showed that a bioactive fraction of *S*. *crispus* leaves also synergized with tamoxifen to cause apoptosis of human breast cancer cell lines without damaging non-malignant epithelial cells. The present study aimed to evaluate the antitumor effect of *S*. *crispus* dichloromethane fraction (F3) using N-methyl-N-Nitrosourea (NMU)-induced rat mammary tumor model. Tumor regression was observed in 75% of the rats following 8-week oral administration of F3 with no secondary tumour formation and no signs of anemia or infection. However, no improvement in the liver and renal function profiles was observed. Major constituents of F3 were identified as lutein, 13^1^-hydroxy-13^2^-oxo-pheophytin a, campesterol, stigmasterol, β-sitosterol, pheophytin a and 13^2^-hydroxy-pheophytin a. These compounds however, may not significantly contribute to the antitumor effect of F3.

## Introduction

Faced with the risk of side effects and reduced efficacy of modern chemotherapeutic drugs such as tamoxifen [1–3], cancer patients seek alternative remedies that are believed to be safe and effective, despite no proper or rigorous scientific analyses performed. More appropriately, scientific research on medicinal plants is becoming more significant in recent years with a number of these natural extracts or their bioactive components being shown to exhibit anticancer activities *in vitro* and *in vivo*. An example of such traditional medicinal plants believed to have anticancer and general health-giving properties is *Strobilanthes crispus* (*S*. *crispus*) [4, 5].

*S*. *crispus* belongs to the “Acanthaceae” family that consists of about 250 species of flowering plants native to tropical Asian countries such as Madagascar and the Malay Archipelago [6]. This plant usually grows on river banks or in abandoned fields and is locally known as “pecah kaca” or “jin batu”. It was first recorded and classified by Thomas Anderson (1832–1870) under Spermatophyta (Flowering Plants and Gymnosperms) [7]. Traditionally the leaves of *S*. *crispus* were boiled with water and the filtrates were consumed as herbal tea (fermented and unfermented) for various medicinal purposes [5]. Reports show that *S*. *crispus* herbal tea has anti-proliferative properties against hormone-dependent and-independent human breast cancer cell lines, MCF-7 and MDA-MB-231, respectively [8, 9]. Since then, a number of studies have reported the cytotoxic effects of various extracts of *S*. *crispus* in a variety of human cancer cell lines [10]. Recent studies from our group identified a bioactive fraction of *S*. *crispus* that was capable of inducing caspase-dependent apoptosis of MCF-7 and MDA-MB-231 cells, without affecting the non-malignant breast epithelial cell line, MCF-10A [11]. However, there are no available reports on the effect of *S*. *crispus* on experimental mammary tumors.

A variety of phytochemicals have been reported in the leaves of *S*. *crispus*. The essential oil of the plant contains mainly phytol, α-cardinol, τ-murolol, ledol and eugenol [12]. Non-volatile constituents include *p*-hydroxy benzoic acid, *p*-coumaric acid, caffeic acid, vanilic acid, gentisic acid, ferulic acid, syringic acid, 1-heptacosanol, tetracosanoic acid, α-amyrin, taraxerol, taraxerone, stigmasterol, β-sitosterol, stigmasterol β-D-glucopyranoside and a rare lignan 4-acetyl-2,7-dihydroxy-1,4,8-triphenyloctane-3,5-dione [13–15]. HPLC analysis also indicated the presence of (+)-catechin, (−)-epicatechin, rutin, myricetin, luteolin, apigenin, naringenin and kaempferol aglycones in the extract of *S*. *crispus*, obtained by supercritical fluid extraction [16]. Although many compounds have been identified thus far from the plant, the active constituents contributing to the anticancer properties of *S*. *crispus* are still unknown.

Chemical-induced mammary tumour rat model is a standard laboratory model for the study of breast cancer because of its biological similarity with human breast cancer in relation to the epithelial origin, malignant features, response to anti-estrogen therapy and gene expression profiles [17, 18]. The chemical carcinogens most commonly used for breast cancer induction in the rodents are N-methyl nitrosourea (NMU) and 7, 12-dimethyl-benzanthracene (DMBA). Rat mammary tumours induced by NMU are particularly known to be estrogen-dependent [19].

Thus the present work was undertaken to determine the effect of an active fraction of *S*. *crispus* on NMU-induced mammary tumors in female Sprague Dawley rats. The bioactive chemical constituents in *S*. *crispus* leaves were also isolated and identified through a systematic *in vitro* anticancer activity-guided approach.

## Materials and Methods

### Chemicals

Hexane, dichloromethane (DCM), chloroform (CHCl_3_), ethyl acetate (EtOAc) and methanol (MeOH) used for extraction and purification of the plant materials were of analytical grade. Silica gel 60 (0.040–0.063 mm) was used for column chromatography. All chemicals used were purchased from Merck (Darmstadt, Germany). NMU and corn oil were purchased from Sigma Chemicals Company (St. Louis, MO, USA). Sodium phenobarbitone was from Alfasan International (BV, Woerden, Holland). Ethanol and xylene were purchased from Teraslab Scientific (BDH Chemicals, Dawsonville, USA).

### Plant Material and Extract Preparation

The leaves of *S*. *crispus* (L.) Blume were purchased from Agrodynamic Resources (Malaysia) who acquired the plant from a cultivated source in Tasek Gelugor, Pulau Pinang in Malaysia. The plant was authenticated by Mr Shunmugam Vellosamy, School of Biological Sciences, Universiti Sains Malaysia (USM). A voucher specimen of the plant (No. 11046) was prepared and deposited at the herbarium in the premise. The plant is not an endangered or protected plant species nor does the collection of this plant violate any international or national rules on biodiversity rights.

Freeze-dried leaves of *S*. *crispus* were pulverized using a mill grinder. The dried powder material was sequentially extracted with a series of organic solvents, starting with hexane, followed by DCM and MeOH in an ultrasonic cleaning bath (42 kHz, 185 W) for 20 min followed by an overnight soak. The extract obtained with each solvent was filtered and the solvent was evaporated *in vacuo* at a temperature below 35°C to yield dried hexane, DCM and MeOH extracts. Among the three extracts, DCM extract that showed significant cytotoxic effect towards MCF-7 and MDA-MB-231 breast cancer cell lines, was subjected to further purification.

The extract (25 g) was chromatographed on silica gel 60 (220 g) using dry vacuum liquid chromatographic technique. Step gradient elution was carried out with hexane: CHCl_3_-EtOAc-MeOH (2:3:0:0 to 0:0:9:1, v/v/v/v) and eluent was collected in fractions of 300 ml. Fractions with similar thin-layer chromatography (TLC) profile were combined to afford five combined fractions, designated as F1 to F5. The cytotoxic activity of all five fractions was then determined using lactate dehydrogenase assay (LDH). The most active fraction, i.e., F3 was subjected to *in vivo* anticancer studies and also further characterized.

### Cell Culture and Cytotoxicity Assay

Breast cancer cell lines MCF-7 and MDA-MB-231 were purchased from the American Type Culture Collection (ATCC) (Rockville, USA). MCF-7 was cultured in Roselle’s Park Memorial Institute (RPMI) 1640 medium while MDA-MB-231 was grown in Dulbecco’s Modified Eagle’s Medium (DMEM). Both media were supplemented with 10% fetal bovine serum and 100 units/mL penicillin-streptomycin. The cell lines were maintained at 37°C in a humidified CO_2_ incubator with 5% CO_2_ in air.

Cytotoxic activity of the plant fractions and the isolated compounds was determined based on the leakage of LDH into the culture supernatant, using the LDH Cytotoxicity Detection Kit (Roche Diagnostics, Germany). The procedure was carried out according to the manufacturer’s recommendation. Cells were seeded in 24-well culture plates at a density of 2 × 10^5^ cells/ml for MCF-7 and 1 × 10^6^ cells/ml for MDA-MB-231 and allowed to attach overnight prior to treatment. The control cultures received <0.1% dimethyl sulfoxide.

### Animals and Experimental Design

Inbred female Sprague Dawley rats (n = 15) were obtained at 36 days of age (body weight 130 g—200 g) from the Animal Research and Service Centre, USM. The animals were housed (in pairs) in polycarbonate cages and acclimatized to standard vivarium conditions (temperature 23 ± 2°C, relative humidity 70 ± 5%, and 14 h light—10 h dark cycle) for 1 week before the commencement of experimental procedures. Standard rodent pellets (Gold Coin Feedmills Sdn. Bhd. Malaysia) and tap water were freely accessible *ad libitum* to the animals throughout the study. Animal beddings (Chipsi, Rosenberg, Germany) were changed regularly, twice per week. Environmental enrichment such as improvised cardboard paper towels for chewing, improvised hard plastic tunnel and balls for exercise were occasionally provided to the animals. This study was approved by the USM Animal Ethics Committee [Ethical approval no. USM/AEC/2011/(69)(304)]. The measures taken to minimize unnecessary pain and distress on the animals include utilization of small sample size, adequate housing and care, short term treatment, use of purely inbred strain to minimize variability of data, frequent monitoring of the animals with high chances of tumor ulceration and early humane end point for the morbid animals among others, all of which are in keeping with the principle of humane experimental technique [20]. Animals were euthanized at the end of the study by using sodium pentobarbital anesthesia. All sections of this study followed the ARRIVE guidelines for reporting animal research [21]. A completed ARRIVE guidelines checklist is included as supplementary information (S1 Fig).

Mammary tumors were induced in the animals with NMU following overnight fasting according to the methods of Thompson *et al*. [22] and Rivera *et al*. [23] with some modifications. Briefly, NMU was administered intraperitoneally in three weekly doses of 50 mg/kg body weight starting from 43 days of age. NMU was freshly dissolved (25 g/ml) in warm normal saline, adjusted to pH 4.0 using 3% acetic acid and maintained at 35–40°C prior to administration. Age-matched control rats were injected with an equivalent volume of the vehicle only.

The animals were weighed weekly following NMU administration. After 3 weeks of the first NMU injection their mammary glands were palpated twice per week for detection of tumors. Palpable tumors started to develop within 6–9 weeks of induction and their locations were recorded. When the tumor size reached 10 mm animals were randomly assigned into untreated (n = 5) and F3-treated (n = 5) groups. A group of uninduced rats (n = 5) was used as normal control. The animals were treated with F3 (40 mg/kg BW) by gavage once daily for eight weeks. Response to the treatment was assessed weekly by the measurement of animal body weight and physical tumor growth parameters (tumor number, size and volume). Tumor multiplicity (average number of tumors per rat) and average tumor volume per group were assessed every two weeks until completion of treatment.

### Tumor Volume Measurement

Changes in the tumor volume were monitored weekly using a digital Vernier caliper. The tumor length (a) and width (b) (in millimeter) were measured at right angles, and the tumor volume (mm^3^) was estimated using Carlson’s formula V = ab^**2**^/2 [24] where ‘a’ and ‘b’ represent the longest and shortest tumor diameters, respectively.

### Blood Collection and Preparation

Animals were euthanized after eight weeks of treatment or earlier due to high tumor burden, using sodium phenobarbitone (50 mg/kg ip). Blood was collected by cardiac puncture and aliquoted into dipotassium ethylenediamine tetra-acetic acid (K_2_EDTA) vials for assessment of full blood count (FBC) and into plane tube gels (SST II advance) for collection of serum used for the assessment of parameters for hepatic or renal injury.

### Measurement of Hematological Parameters

FBC parameters were assessed using automated hematology analyzer XE-5000 (Sysmex Asia Pacific, Pte Ltd, Singapore) according to the manufacturer’s guidelines. The parameters measured include total red blood cell (RBC) count, hemoglobin (Hb) content, packed cell volume (PCV) and red cell indices [i.e., mean corpuscular volume (MCV), mean corpuscular hemoglobin (MCH) content, mean corpuscular hemoglobin concentration (MCHC) and red cell distribution width (RDW)]. Total leukocyte count, differential leukocyte (neutrophils, lymphocytes, monocytes, eosinophils, basophils) counts and platelet (PLT) count were also measured.

### Measurement of Biochemical Parameters

Biochemical parameters for liver and kidney function impairment were analyzed using automated chemistry analyzer (Architect c8000; Abbott Park, USA) according to the manufacturer’s guidelines. The parameters measured were serum total protein (TP), alkaline phosphatase (ALP), aspartate aminotransferase (AST), alanine aminotransferase (ALT), urea, creatinine, uric acid, calcium and electrolytes.

### Isolation of Chemical Constituents

F3 was chromatographed on silica gel by flash column chromatography. Approximately 2 g of F3 was loaded onto the column. The mobile phase was made up of a gradient composition of EtOAc in CHCl_3_ (4 to 100%). A total of six sub fractions were obtained. From there, the third sub fraction was repeatedly chromatographed on silica gel preparative-TLC using the mobile phase that consisted of CHCl_3_-EtOAc (4:1, v/v) to afford compound **1** which was obtained as yellowish-orange crystals and compound **2** which was a greyish green powder. Chromatography of the second sub fraction of F3 on silica gel 60 using an isocratic mobile phase of hexane-CHCl_3_-EtOAc (3:5:2, v/v/v) yielded white solid that consisted of a mixture of compounds **3**, **4** and **5**.

Compounds **6** and **7** present in F3 were also found to be present in higher abundance in the preceding fraction, F2. Hence, in order to get adequate yield of both compounds for further work, isolation of these two compounds was carried out from F2. The fraction (2 g) was subjected to flash column chromatography using a step gradient of hexane-EtOAc (1:0 to 0:1, v/v) to yield twenty sub fractions. The eleventh sub fraction was further purified by silica gel flash column chromatography using increasing amount of CHCl_3_ in hexane (0 to 100%) and subsequently developed twice on silica gel preparative-TLC using hexane-CHCl_3_-EtOAc (5:4:1, v/v/v) and hexane-CHCl_3_-EtOH (5:4.5:0.5, v/v), respectively, to afford the two pure compounds.

### Identification and Structure Elucidation of Compounds

Structure elucidation of compounds **1, 2, 6** and **7** was done by nuclear magnetic resonance (NMR) spectroscopy and mass spectrometry (MS). NMR was carried out on Avance 500 NMR spectrometer (Bruker, Germany) operating at 25°C. The samples were dissolved in deuterated chloroform (CDCl_3_) and the chemical shifts were recorded with reference to that of tetramethylsilane δ (0, ppm). Mass spectrometry (MS) was carried out using AmaZon X mass spectrometer for nominal mass (Bruker, Germany). Accurate mass analysis was acquired using MicroTOF-Q II 10269 system (Bruker, Germany). Compound ionization was achieved by electrospray ionization (ESI) in the positive mode. Identification of compounds **3–5** was done through hyphenated gas chromatography mass spectrometry (GC-MS) (Agilent, Germany). Separation was achieved on a HP-5MS column, 30 m × 0.25 μm, 0.25 m, with helium gas as the carrier at a flow rate of 0.5 ml/min. Mass acquisition was performed in the range of 40–550 a.m.u. using electron impact ionization at 70 eV. Compound identity was determined by matching the mass spectra of each compound with the MS data in the National Institute of Standards and Technology (NIST) database. Complete NMR spectra of all seven isolated compounds are provided in S2 Fig.

### Statistical Analysis

Data were analyzed using SPSS Version 20 (IBM Corp, USA) and results were expressed as median and interquartile range (IQR). Repeated measure analysis of variance (ANOVA) was used for comparison of average tumor number and tumor volume between F3-treated and untreated groups while significant differences in liver and kidney function parameters were computed using Mann-Whitney U-test. p < 0.05 was considered statistically significant.

## Results

### Cytotoxic Activity of *S*. *crispus* Extracts and DCM fractions

The ability of hexane, DCM and MeOH extracts of *S*. *crispus* to induce cell death was evaluated on MDA-MB-231 and MCF-7 breast cancer cells. At the test concentration of 100 μg/ml, DCM extract was significantly cytotoxic towards both MDA-MB-231 and MCF-7 cells with approximately 50% and 63% growth inhibition within 48 h, respectively (Table 1). The hexane and methanol extracts were not cytotoxic to both types of cells.

**Table 1:** Cytotoxic activity of various extracts of *S*. *crispus* leaves.

Extract	Cytotoxicity (%)	
	MDA-MB-231	MCF-7
Hexane	2.2 ± 0.1	2.5 ± 0.8
DCM	*47.0 ± 7.6	*63.2 ± 10.1
MeOH	4.8 ± 1.6	2.2 ± 2.0

MDA-MB-231 and MCF-7 cells were treated with 100 μg/ml of the extracts. Cell death was determined using the LDH assay. Statistical analysis was performed using Kruskal Wallis

*p < 0.05

**Fig 1:**
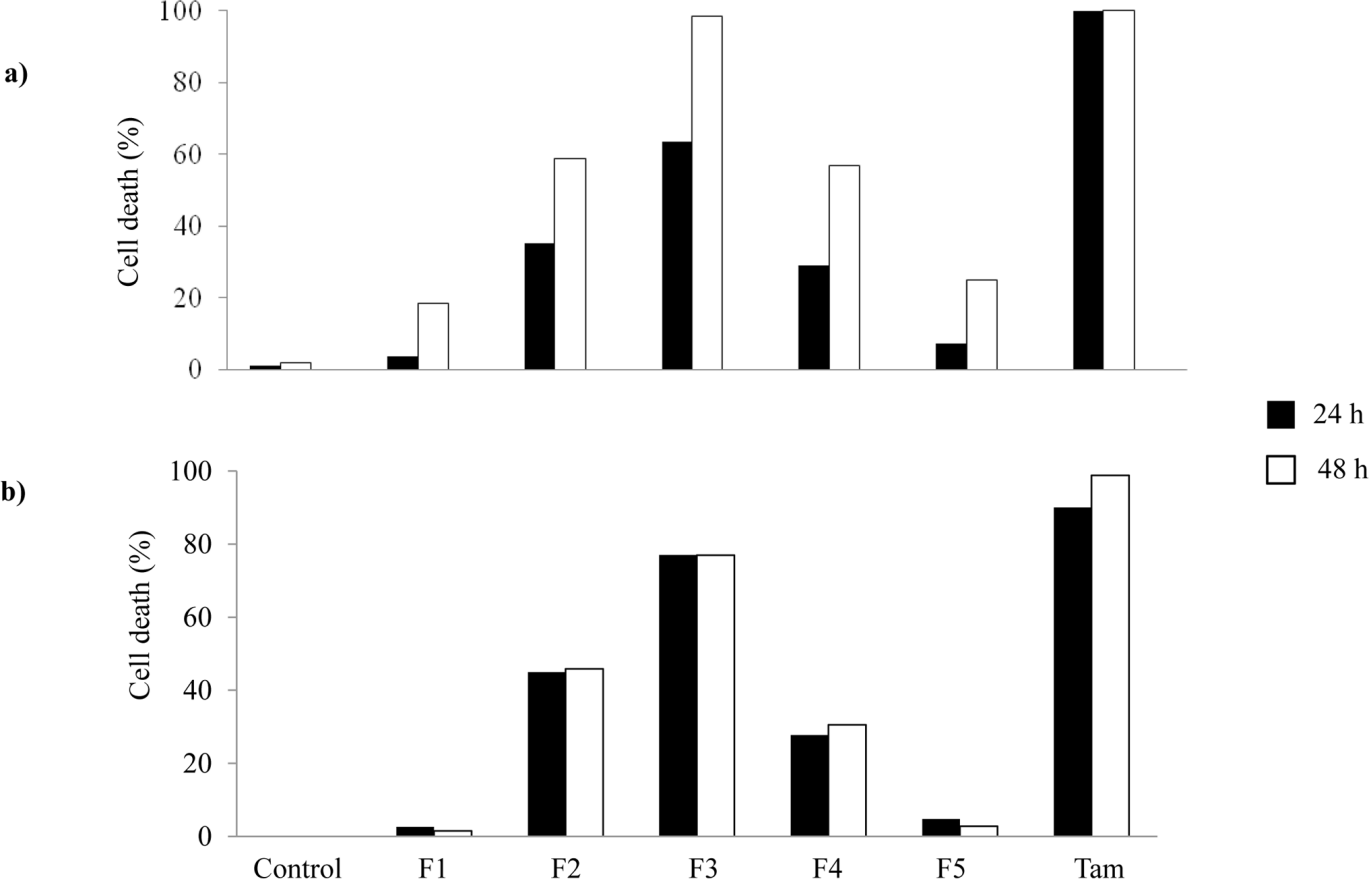
Cytotoxic activity of various fractions of *S*. *crispus* DCM extract in breast cancer cell lines. MDA-MB-231 (a) and MCF-7 (b) cells were treated with 100 μg/ml fractions (F1-F5) for 24 and 48 h. Percentage cell death was determined using the LDH assay and 15 μM tamoxifen (Tam) was used as the positive control.

The DCM extract was further chromatographed into five fractions, namely F1 to F5 and their cytotoxic activity was evaluated. Cytotoxicity was found to increase drastically from F1 to F3 and then decreased in F4 and F5 (Fig 1). This indicated that the bioactive constituents in *S*. *crispus* were of medium polarity since F2, F3 and F4 were eluted with mid polar solvents. The cytotoxic action of F1 to F5 in MDA-MB-231 cells was time-dependant as cell death was found to increase by 30–50% from a treatment period of 24 h to 48 h (Fig 1A). On the other hand, maximum cytotoxicity was found against MCF-7 cells by 24 h post-treatment (Fig 1B). The most active fraction was F3 that caused almost 100% cell death in MDA-MB-231 cells at 48 h and slightly below 80% cell death in MCF-7 cells at both 24 and 48 h. Further analysis was carried out on F3 to evaluate the chemical constituents in this fraction.

### Effects of F3 on the End Tumor Burden and Animal Body Weight

**Fig 2:**
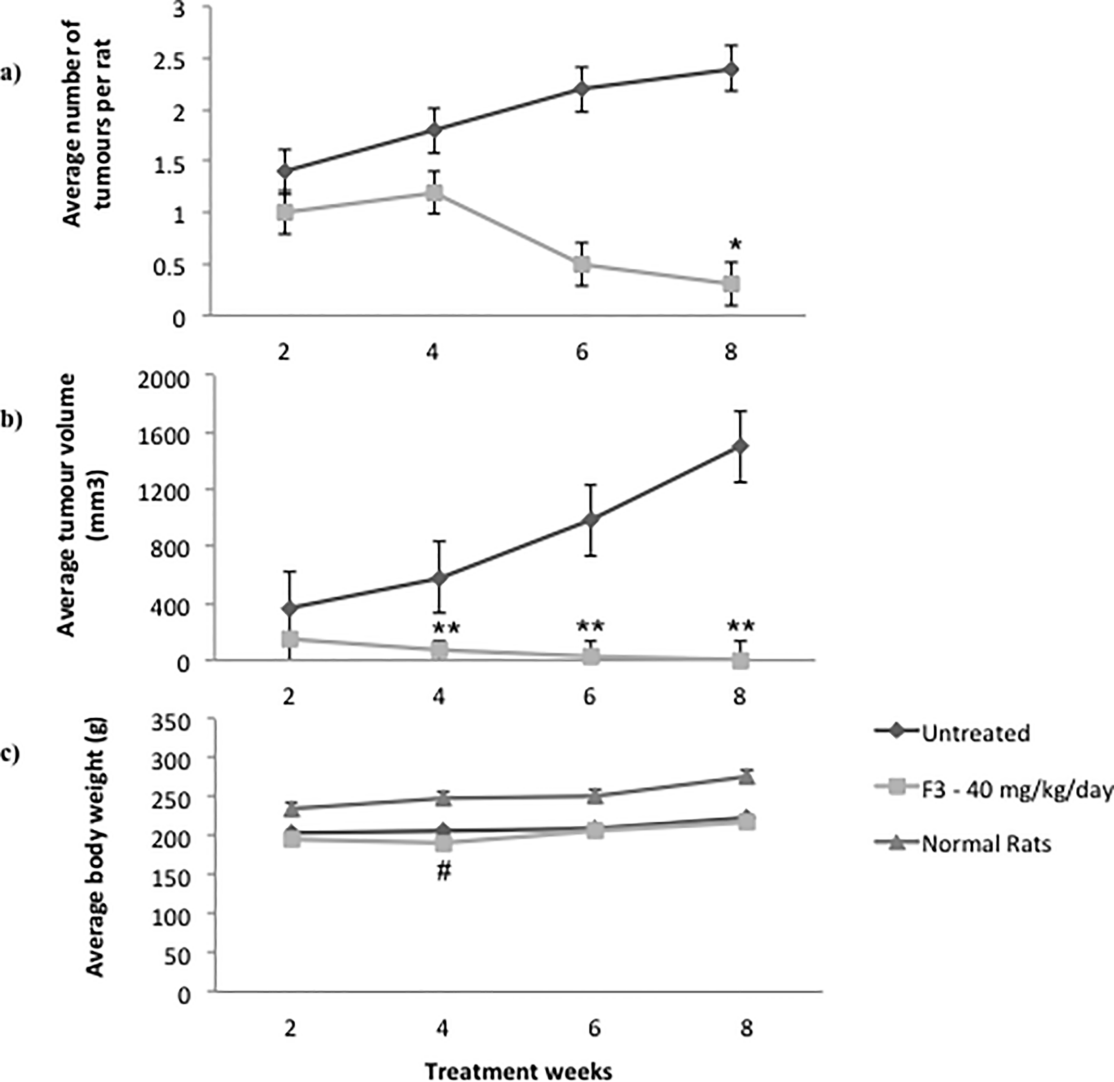
Effects of F3 on the end-tumor burden and body weight. Tumor multiplicity (a), average tumor volume (b) and body weight (c) were repeatedly measured at different time points following treatment with F3 (40 mg/kg/day). P values were calculated using Mann-Whitney test for comparison of end tumor physical parameters between untreated (n = 5) and F3-treated (n = 4) rats. *p < 0.05, **p < 0.01 compared to untreated group. ^#^p < 0.05 compared to normal controls (n = 5).

Fig 2 shows time-dependent changes in tumor number and tumor volume in F3-treated and untreated tumor-bearing animals. One of the five animals treated with F3 developed ulceration of the tumor nodule that subsequently became infected after 6 wks of treatment. This animal was therefore sacrificed earlier and excluded from the analysis. Complete regression of mammary tumors was observed in 75% of the animals treated with F3 and partial tumor regression in the rest compared to the untreated control (p < 0.05). Complete tumor regression refers to the absence of palpable tumor nodule after eight weeks of treatment. Consequently, the average final tumor volume reduced significantly in this group (p < 0.01) starting from the fourth week of treatment (Fig 2). However, the mean final tumor weight at necropsy (0.00 ± 3.45) was not significantly different from the untreated tumor-bearing animals (1.54 ± 18.05). Although there was incidental significant loss of body weight after F3 administration compared to normal controls, the values were similar to the untreated group. No secondary tumor formation and no significant complication developed in any of the animals throughout the study. Thus, administration of F3 was significantly effective in reducing the overall tumor burden compared to the untreated tumor-bearing animals.

### Effects of F3 on the Hematological Parameters

FBC results (Table 2) show no significant derangement due to F3 administration compared to normal values except for the low MCH and high platelet count that indicate anemia and transient inflammation possibly due to the effect of carcinogen administered or other host factors. Notably, decreased red cell mass parameters (total RBC, Hb, and PCV), marginal elevation of red cell size parameters (MCV, MCH, and RDW) and increased total leucocyte count which suggest macrocytic anemia and secondary bacterial infection in the untreated tumor-bearing rats, were significantly modulated by F3 (p <0.05), while the mean Hb concentration and RDW were improved towards normal values (although not statistically significant). Incidentally, there were low lymphocyte counts, marginally elevated monocyte and high polymorph differential counts in the animals treated with F3 compared to the normal controls.

**Table 2:** FBC parameters in normal, untreated and F3-treated animals.

Parameters	Tumour untreated (n = 5)	F3-treated (40 mg/kg, n = 4)	Normal rats (n = 5)
Hemoglobin (g/dl)	13.2 (1.3)	13.8 (1.9)	14.1 (1.0)
Packed cell volume (%)	41.0 (0.02)	47.0 (0.03)	44.0 (0.02)
Red Blood Cells (x 10^12/L)	6.4 (0.7)	7.3 (0.8)	7.10 (0.5)
Mean Corpuscular Volume (fl)	66.0 (3.0)	61.5 (3.0)	62.0 (1.0)
Mean Corpuscular hemoglobin (pg)	21.0 (2.0)	18.0 (1.0)*	19.5 (1.0)
Mean Corpuscular hemoglobin concentration (g/L)	310.0 (5.0)	315.0 (18.0)	320.0 (25.0)
Red cell distribution width (%)	12.9 (0.4)	11.8 (1.7)	11.7 (0.7)
White Blood Cells (x 10^9/L)	3.5 (4.8)	1.8 (1.3)	1.9 (1.2)
Platelets (x 10^9/L)	716.0 (157.0)	767.0 (57.0)*	687.0 (188.0)

P values were calculated using the Mann-Whitney test for categorical data between normal and F3-treated rats. Results are presented as median values with IQR in brackets.

*p < 0.05 compared to normal rats

### Effects of F3 on Liver Function

Serum levels of liver enzymes, ALT and AST, were low in the untreated animals but were high after eight weeks administration of F3 compared to the normal controls (Table 3). The differences are however, not statistically significant due to high IQR values. Nevertheless, when F3-treated animals were compared with the untreated tumor-bearing group, there was an overall improvement of liver function towards normal values. Thus, F3 generally has beneficial effects although it may also contain substances with potential side effects on the liver function but this has yet to be confirmed.

**Table 3:** LFT parameters in normal, untreated and F3-treated animals.

Parameters	Tumour untreated (n = 5)	F3-treated (40 mg/kg, n = 4)	Normal rats (n = 5)
Total protein (g//L)	65.80 (8.8)	68.0 (2.9)	71.20 (5.4)
Aspartate aminotransferase (U/L)	170.0 (112.0)	222.0 (57.0)	205.0 (85.0)
Alanine aminotransferase (U/L)	35.0 (26.0)	76.0 (43.0)	51.0 (35.0)
Alkaline phosphatase (U/L)	156.0 (154.0)	130.0 (107.0)	148.0 (50.0)

P values were calculated using the Mann-Whitney test for categorical data between normal and F3-treated rats. Results are presented as median values with IQR in brackets.

### Effects of F3 on Renal Function

NMU administration induced some derangement of serum electrolytes with low creatinine, potassium, urea and uric acid levels compared to the normal values (Table 4). Eight-week treatment of the tumor-bearing animals with F3 showed some improvement in the RFT profile with creatinine and uric acid values being increased towards normal levels. However, the serum chloride concentration was significantly higher than the normal control (p <0.05) suggesting some impairment in renal function.

**Table 4:** RFT parameters in normal, untreated and F3-treated animals.

Parameters	Tumour untreated (n = 5)	F3-treated (40 mg/kg, n = 4)	Normal rats (n = 5)
Sodium (mmol/l)	142.0 (2.0)	142.5 (1.9)	141.4 (2.6)
Creatinine (mmol/l)	42.24 (3.9)	45.5 (3.6)*	51.0 (3.9)
Potassium (mmol/l)	4.6 (0.8)	4.4 (0.8)*	5.5 (0.8)
Chloride (mmol/l)	99.0 (7.0)	103.5 (3.0)*	99.0 (4.0)
Urea (mmol/l)	7.3 (2.2)	7.0 (1.0)*	9.0 (1.6)
Uric Acid (μmol/l)	95.7 (24.0)	128.6 (23.9)	131.6 (62.8)
Calcium (μmol/l)	2.9 (1.1)	2.4 (0.2)*	2.7 (0.3)

P values were calculated using the Mann Whitney test for categorical data between normal and F3-treated rats. Results are presented as median values with IQR in brackets.

*p < 0.05 compared to normal rats

### Identification of the Chemical Constituents in F3

**Fig 3:**
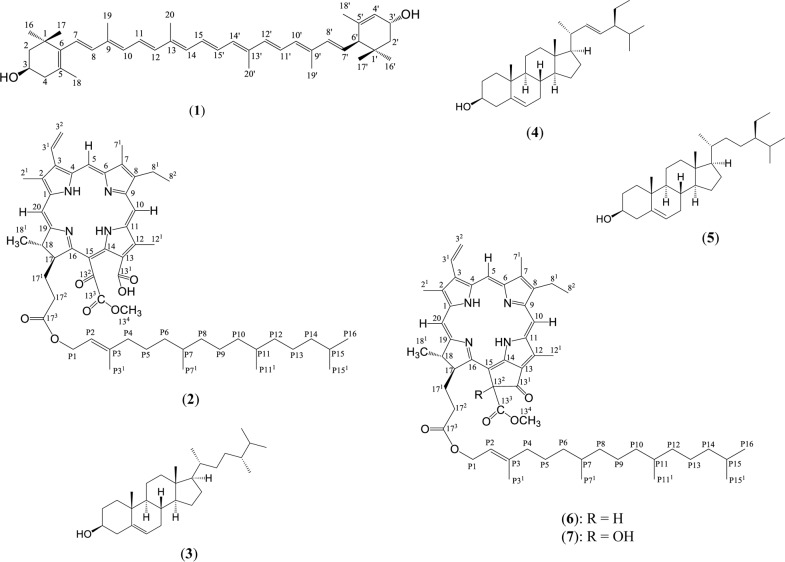
Chemical structures of compounds isolated from F3. (1) lutein (2) 13^1^-hydroxy-13^2^-oxo-pheophytin a (3) campesterol (4) stigmasterol (5) β-sitosterol (6) pheophytin a (7) 13^2^-hydroxy-pheophytin a.

In order to gain a better insight on the chemical composition of F3 and the contribution of the chemical constituents to its anticancer activity, we proceeded to isolate and identify the main chemical compounds in this fraction. Following multiple steps of chromatographic procedures, seven major compounds were identified (Fig 3). The first compound was found to be lutein based on the following spectroscopic data and comparison with the data in the literature [25–27].

Lutein (**1**): Yellowish-orange crystals. ESI-MS molecular ion peak at m/z 568.3 [M]^+^ corresponding to C_40_H_56_O_2_. ^1^H NMR (500MHz, CDCl_3_): δ 6.65 (4H, *m*, H-11, H-11’, H-15, and H-15’), 6.36 (2H, *d*, *J* = 14.9, H-12 and H-12’), 6.27 (2H, *d*, *J* = 9.7, H-14 and H-14’), 6.14 (5H, *m*, H-7, 8, 8’, 10 and 10’), 5.54 (1H, *bs*, H-4’), 5.43 (1H, *dd*, *J* = 15.5 and 9.9 Hz, H-7’), 4.25 (1H, *m*, H-3’), 4.00 (1H, *m*, H-3), 2.40 (1H, *bd*, *J* = 9.9 Hz, H-6’), 2.39 (1H, *dd*, *J* = 17.1, 5.2 Hz, H-4a), 2.05 (1H, *dd*, *J* = 17.1, 10.1 Hz, H-4b), 1.97 (3H, *s*, H-19)*, 1.96 (6H,*s*, H-20, H-20’)*, 1.91 (3H, *s*, H-19’), 1.84 (2H, *dd*, *J* = 13.2, 5.9 Hz, H-2’a), 1.77 (2H, *ddd*, *J* = 12.0, 3.3, 2.1 Hz, H-2), 1.74 (3H, *s*, H-18), 1.62 (3H, *s*, H-18’), 1.48 (2H, *t*-like, *J* = 12.0 Hz, H-2), 1.36 (2H,*dd*, *J* = 13.2, 6.9Hz, H-2’b), 1.07 (6H, *s*, H-16 and H-17), 1.00 (3H, *s*, H-17’), 0.85 (3H, *s*, H-16’). ^13^C NMR (125MHz, CDCl_3_): 138.51 (C-8), 138.01 (C-6), 137.77 (C-5’), 137.74 (C-12), 137.58 (C-8’)^a^, 137.57 (C-12’)^a^, 136.51 (C-13)^b^, 136.43 (C-13’)^b^, 135.71 (C-9), 135.09 (C-9’), 132.6 (C-14, 14’), 131.32 (C-10)^c^, 130.82 (C-10’)^c^, 130.10 (C-15)^d^, 130.05 (C-15’)^d^, 128.75 (C-7’), 126.18 (C-5), 125.60 (C-7), 124.95 (C-11), 124.8 (C-11’), 124.49 (C-4’), 65.9 (C-3’), 65.1 (C-3), 54.97 (C-6’), 48.43 (C-2), 44.66 (C-2’), 42.56 (C-4), 37.14 (C-1), 34.05 (C-1’), 30.28 (C-17), 29.51 (C-17’), 28.74 (C-16), 24.28 (C-16’), 22.89 (C-18’), 21.64 (C-18), 13.13 (C-19’), 12.83 (C-20, C-20’), 12.78 (C-19). ^a-d^Assignments of resonances may be interchanged between those labelled with the same alphabet.

The second compound was isolated as a greyish green solid. HR-ESI-MS analysis in the positive mode showed the molecular ion mass of m/z 903.5630 [M+H]^+^ corresponding to the molecular formula of C_55_H_75_N_4_O_7_ with 21 degree of unsaturation. Based on its red fluorescence appearance on TLC under UV long wave and its high degree of unsaturation, this compound was considered to be a porphyrin (chlorophyll related compound). ^1^H NMR spectrum of the compound exhibited three downfield singlets at δ 9.76, 9.56 and 8.70 ppm corresponding to three aromatic methine protons of the porphyrin located at H-10, H-5 and H-20, respectively. Three sets of coupling doublet of doublets at δ 8.02, 6.34 and 6.17 ppm that had a correlation with the quarternary carbon at δ 136.55 ppm in HMBC were characteristic of the exocyclic ethylene group attached to C-3. The presence of four isolated methyl protons at H-12^1^, H-13^4^, H-2^1^ and H-7^1^ were indicated by four singlets at δ 3.90, 3.74, 3.44 and 3.28 ppm, respectively. Their positions were determined based on HMBC data. A pair of coupling signals made up of a quartet (δ 3.75 ppm) and a triplet (δ 1.72 ppm) that correlated to the carbon at δ 146.00 ppm in HMBC was typical of an ethyl group substituted on C-8. The presence of two coupling upfield methine proton multiplets at δ 5.14 (H-17) and δ 4.44 ppm (H-18) indicated that the structure assumed a chlorin moiety whereby one of the pyrrolic ring (ring D) is saturated at the δ position (positions 17 and 18). The doublet at δ 1.63 ppm which corresponded to the methyl protons H-18^1^, four multiplets in the region of δ 2.4–2.8 ppm that belonged to two pairs of diastereotopic protons H-17^1^a & b and H-17^2^a & b which coupled to the carbonyl carbon at δ 173.32 ppm (C-17^3^), together with a large number of overlapping proton signals in the region of δ 1.6–0.7 ppm belonging to the aliphatic methylene and methyl functions of phytyl further indicated that the structure was close to that of pheophytin a (**6**). However, the absence of H-13^2^ signal and the significant downfield shift of C-13^2^ signal to δ 161.04 ppm in compound **2** indicated that the 13^2^ position in this compound consisted of a carbonyl group and a C = OCOOCH_3_ functionality must exist. Furthermore, to match the molecular formula, an additional OH group should exist in compound **2**. This hydroxyl group was most probably attached to C-13^1^ to form a COOH functionality so that the compound has a cleaved E ring structure as shown in Fig 3. The NMR data is provided in Table 5 for future reference as no NMR data of this compound is available in the literature. Based on all the spectroscopy data above, compound **2** was identified as 13^1^-hydroxy-13^2^-oxo-pheophytin a (synonym: purpurin 7-monomethyl phytyl ester).

**Table 5:** ^1^H-NMR (500 MHz), ^13^C NMR (125 MHz) and 2D NMR data of 13^1^-hydroxy-13^2^-oxo-pheophytin a (2) in CDCl_3_.

Position	^1^H (m, *J*)	^13^C	HMBC (C→H#)	COSY
**Chlorine ring**				
1	-	141.22	-	-
2	-	131.44	-	-
2^1^	3.44 (s)	12.13	1, 2, 3	-
3	-	136.06	-	-
3^1^	8.02 (dd, 11.9; 17.8)	129.00	2, 3, 3^2^, 4	3^2^a, 3^2^b
3^2^	6.34 (dd,1.3; 17.8)	122.75	3, 3^1^	3^1^, 3^2^b
	6.17 (dd, 1.3; 11.9)		2, 3, 3^1^	3^1^, 3^2^a
4	-	136.55	-	-
5	9.56 (s)	99.66	4	-
6	-	131.52	-	-
7	-	145.60	-	-
7^1^	3.28 (s)	11.32	6, 7, 8	-
8	-	149.72	-	-
8^1^	3.75(q, 7.6)	19.73	7, 8, 9	8^2^
8^2^	1.72 (t, 7.6)	19.66	8, 8^1^	8^1^
9	-	142.87	-	-
10	9.76 (s)	104.15	8, 11, 12	-
11	-	130.27	-	-
12	-	141.22	-	-
12^1^	3.90 (s)	12.46	11, 12, 13	-
13	-	101.96	-	-
13^1^	-	150.02	-	-
13^2^	-	161.04	-	-
13^3^	-	171.15	-	-
13^4^	3.74 (s)	54.16	13^3^	-
14	-	111.34	-	-
15	-	100.45	-	-
16	-	166.33	-	-
17	5.14 (m)	53.70	18^1^	17^1^a & 17^1^b
17^1^	2.81 (m)	24.77		17
	2.05 (m)			17
17^2^	2.34 (m)	32.13	17^3^	-
	2.16 (m)		17^3^	17^2^b
17^3^	-	173.32	-	-
18	4.44 (m)	50.16	16, 18^1^	18^1^
18^1^	1.63 (d)	22.72	18	18
19	-	170.90	-	-
20	8.70 (s)	93.89	18	-
NH	-1.07 (s); -1.36 (s)			-
*Phytyl side chain*				
P1	4.44 (m)	61.49		P2
P2	5.14 (t, 7.6)	117.74		P1
P3	-			
P3^1^	1.57 (s)	16.23		-
P7^1^	0.77 (d, 6.6)*	19.63**		-
P11^1^	0.80 (d, 6.6)*	19.57**		-
P15	1.29			
P15^1^	0.85 (d, 6.6)	22.62		P-15
P16	0.85(d, 6.6)	22.62		P-15
P4-P14	1.6–0.9			

*, ** Assignments may be interchanged

Another three compounds isolated as a mixture from F3 were campesterol (**3**), stigmasterol (**4**) and β-sitosterol (**5**). The identity of these compounds was determined by GC-MS analysis and matching of the mass spectral data with that in the NIST database. The MS data of these compounds are given in Table 6. The sixth and seventh compounds were greenish pigments that are structurally related to chlorophyll. Based on the following spectroscopic data and comparison with the data in literature [28, 29], these compounds were identified as pheophytin (**6**) and 13^2^-hydroxy-pheophytin a (**7**)

**Table 6:** GC-MS data for compounds 3–5.

Retention time (min)	Mass spectral data, m/z (relative intensity)*	Compound name	Relative composition (%)
20.84	*Obtained*: 400 (M^+^, 60), 43 (100), 382 (40), 315 (40), 55 (39), 367 (38), 289 (37), 145 (37), 213 (36), 107 (35)	Campesterol	15.89
	*NIST Library*: 400 (M^+^, 40), 43 (100), 55 (43), 107 (30), 145 (25), 213 (20), 315 (20), 289 (18), 382 (17), 367 (15)		
21.52	*Obtained*: 412 (M^+^, 90), 55 (100), 81 (60), 255 (58), 159 (45), 133 (40), 300 (38), 107 (36), 351 (35), 379 (20)	Stigmasterol	52.75
	*NIST Library*: 412 (M^+^, 20), 55 (100), 81 (55), 107 (25), 133 (22), 159 (20); 271 (18), 351 (10), 300 (12), 379 (5)		
22.64	*Obtained*: 414 (M^+^, 90), 43 (100), 329 (60), 396 (40), 303 (40), 213 (38), 145 (36), 381 (37), 255 (30), 133 (25)	*β*-Sitosterol	25.93
	*NIST Library*: 414 (M^+^, 35), 43 (100), 145 (25), 133 (20), 213 (18), 255 (17), 303 (18), 329 (27), 396 (17), 381 (15)		

* Major and representative m/z values only

Pheophytin a (**6**): Dark green powder. ESI-MS molecular ion peak at m/z 871.5 [M+H]^+^ corresponding to C_55_H_75_N_4_O_5_. ^1^H NMR (500MHz, CDCl_3_): δ 9.49 (1H, *s*, H-10), 9.35 (1H, *s*, H-5), 8.55 (1H, *s*, H-20), 7.97(1H, *dd*, *J* = 17.8, 11.5 Hz, H-3^1^), 6.27 (1H, *s*, H-13^2^), 6.26 (2H, *dd*, *J* = 17.8, 1.3 Hz, H-3^2^ *E*), 6.16 (2H, *dd*, *J* = 11.5, 1.3 Hz, H-3^2^ *Z*), 5.13 (1H, *t*, J = 7.0 Hz H-P2), 4.49 (1H, *m*, H-18), 4.45, (2H, *m*, H-P1), 4.21 (1H, *dt*, *J* = 8.1, 2.7 Hz, H-17), 3.88 (1H, *s*, H-13^4^), 3.68 (1H, *s*, H-12^1^), 3.67 (3H, *q*, *J* = 7.6 Hz, H-8^1^), 3.39 (3H, *s*, H-2^1^), 3.21 (3H, *s*, H-7^1^), 2.63 (1H, *m*, H-17^1^a), 2.48 (1H, *m*, H-17^2^a), 2.34 (1H, *m*, H-17^1^b), 2.20 (1H, *m*, H-17^2^b), 1.87 (2H, *m*, P4), 1.80 (3H, *d*, *J* = 7.4 Hz, H-18^1^), 1.68 (3H, *t*, *J* = 7.6 Hz, H-8^2^), 1.57 (3H, *s*, H-P3^1^), 1.49 (1H, *m*, H-P15), 0.85 (6H, *d*, *J* = 6.6 Hz, H-P15^1^, H-P16), 0.80 (3H, *d*, J = 6.6 Hz, H-P7^1^), 0.78 (3H, *d*, *J* = 6.6 Hz, H-P11^1^), 1.4–0.9 (H-P5-P14), -1.46 (*s*, NH), -1.65 (*s*, NH). ^13^C NMR (125MHz, CDCl_3_): δ 189.66 (C-13^1^), 173.02, (C-17^3^), 172.23 (C-19), 169.62 (C-13^3^), 161.24 (C-16), 155.60 (C-6), 150.97 (C-9), 149.65 (C-14), 145.21 (C-8), 142.86 (C-P3), 142.03 (C-1), 137.93 (C-11), 136.50 (C-3)^a^, 136.26 (C-4)^a^, 136.16 (C-7)^a^, 131.84 (C-2), 129.08 (C-3^1^)^b^, 129.04 (C-12)^b^, 129.00 (C-13)^b^, 122.71 (C-3^2^), 117.70 (C-P2), 105.21 (C-15), 104.42 (C-10), 97.52 (C-5), 93.12 (C-20), 64.70 (C-13^2^), 61.47 (C-P1), 52.86 (C-13^4^), 51.12 (C-17), 50.11 (C-18), 39.78–24.41 (C-P4-P14), 31.18 (C-17^2^), 29.81 (C-17^1^), 28.00 (C-P15), 23.07 (C-18^1^), 22.71 (C-P16)^c^, 22.54 (C-P15^1^)^c^, 19.71 (C-P11^1^)^d^, 19.62 (C-P7^1^)^d^,19.44 (C-8^1^), 17.42 (C-8^2^), 16.28 (C-P3^1^), 12.12 (C-12^1^), 12.10 (C-2^1^), 11.23 (C-7^1^). ^a-d^Assignments of resonances may be interchanged between those labelled with the same alphabet.

13^2^-hydroxy-pheophytin a (**7**): Greenish dark brown powder. ESI-MS molecular ion peak at m/z: 909.4 [M+Na]^+^ corresponding to C_55_H_74_N_4_O_6_Na. ^1^H NMR (500MHz, CDCl_3_): δ 9.61 (1H, *s*, H-10), 9.47 (1H, *s*, H-5), 8.63 (1H, *s*, H-20), 8.02 (1H, *dd*, *J* = 17.8, 11.6 Hz, H-3^1^), 6.32 (1H, *dd*, *J* = 17.9, 1.2 Hz, H-3^2^ *E*), 6.21 (1H, *dd*, *J* = 11.6, 1.3 Hz, H-3^2^ *Z*), 5.51 (1H, *s*, H-13^2^-OH), 5.21 (2H, *m*, H-P2), 4.56 (1H, *m*, H-P1), 4.41 (1H, *m*, H-18), 4.15 (1H, *m*, H-17), 3.74 (3H, *s*, H-12^1^), 3.71 (3H, *q*, *J* = 7.7 Hz, H-8^1^), 3.61 (1H, *s*, H-13^4^), 3.42 (3H, *s*, H-2^1^), 3.25 (3H, *s*, H-7^1^), 2.94 (1H, *m*, H-17^1^a), 2.55 (2H, *m*, H-17^2^a & 17^2^b), 2.28 (1H, *m*, H-17^1^a), 1.91 (2H, *m*, H-P4), 1.71 (3H, *t*, *J* = 7.7 Hz, H-8^2^), 1.61 (3H, *s*, P3^1^), 1.60 (2H, *d*, *J* = 7.3 Hz, H-18^1^), 1.50 (1H, *m*, H-P15), 1.37–0.9 (H-P4-P14), 0.85 (6H, *d*, *J* = 6.6 Hz, H-P15^1^ & P16), 0.80 (3H, *d*, *J* = 8.5, H-P7^1^)*, 0.79 (3H, *d*, *J* = 8.5Hz, H-P11^1^)*, -1.83 (*s*, NH). ^13^C NMR (125MHz, CDCl_3_): δ 192.02 (C-13^1^), 173.60 (C-17^3^), 172.81 (C-13^3^), 172.43 (C-19), 162.49 (C-16), 155.4 (C-6), 151.06 (C-9), 149.86 (C-14), 145.24 (C-8), 142.78 (C-P3), 142.04 (C-1), 137.83 (C-11), 136.54 (C-7), 136.30 (C-3)^a^, 136.23 (C-4)^a^, 131.77 (C-2), 129.43 (C-12), 129.10 (C-3^1^), 126.98 (C-13), 122.89 (C-3^2^), 117.89 (C-P2), 107.70 (C-15), 104.28 (C-10), 97.90 (C-5), 93.66 (C-20), 88.99 (C-13^2^), 61.57 (C-P1), 53.42 (C-13^4^), 51.85 (C-17), 50.34 (C-18), 39.84–24.44 (C-P4-P14), 31.60 (C-17^2^), 31.16 (C-17^1^), 29.97 (C-P15), 22.72 (C-P16)^b^, 22.66 (C-P15^1^)^b^, 22.63 (C-18^1^), 19.71 (C-P11^1^)^c^, 19.62 (C-P7^1^)^c^, 19.51 (C-8^1^), 17.47 (C-8^2^), 16.34 (C-P3^1^), 12.31 (C-12^1^), 12.13 (C-2^1^), 11.30 (C-7^1^). ^a-d^Assignments of resonances may be interchanged between those labelled with the same alphabet.

### Cytotoxicity of Chemical Constituents of F3

**Fig 4:**
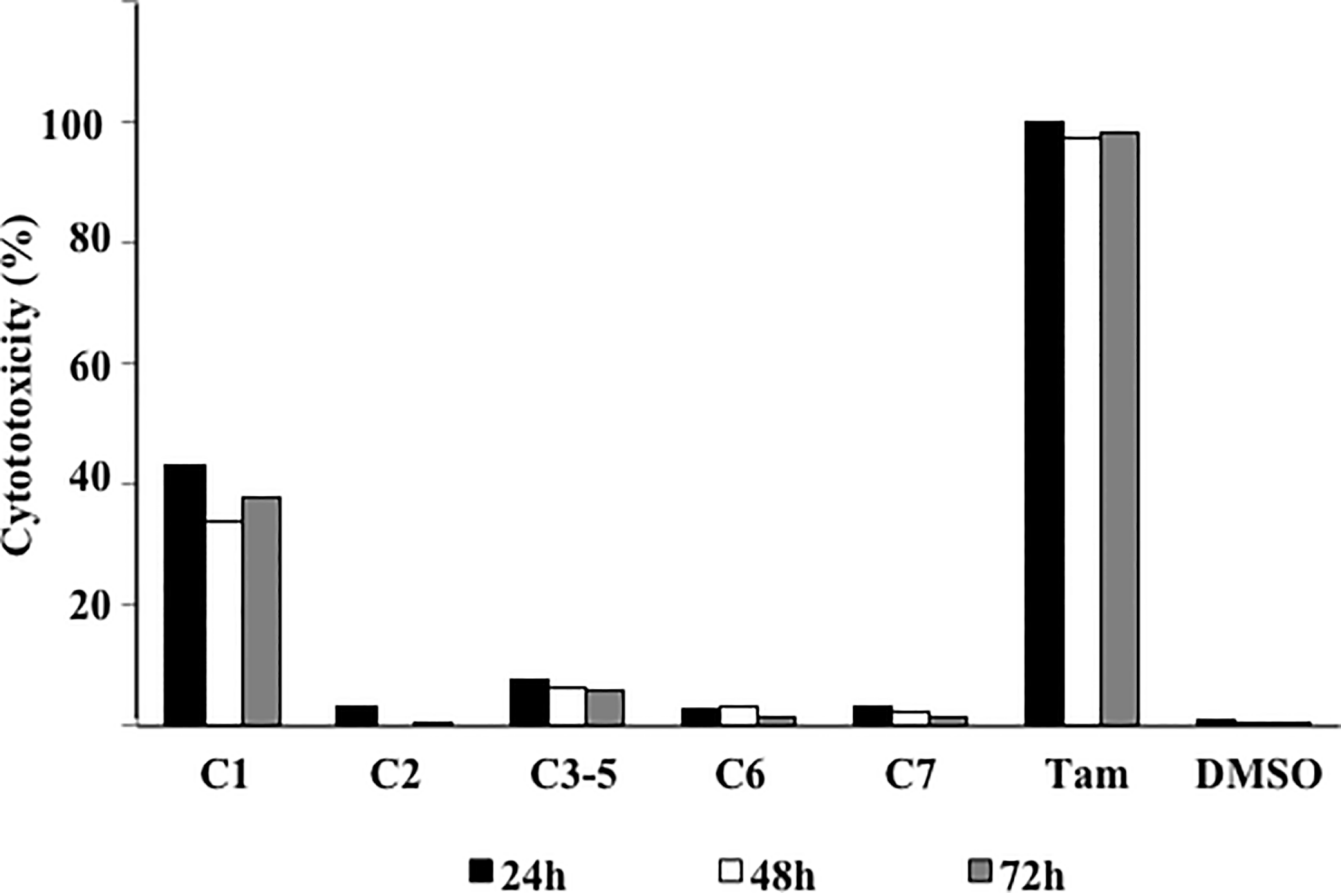
Cytotoxic effect of compounds isolated from F3 on MCF-7 cells. Cells were treated with 100 μM of the compounds (C1-C7) for up to 72 h and cell death was determined by LDH assay. Tamoxifen (15 μM) was used as the positive control.

The compounds isolated from F3 were evaluated for their cyotoxicity against MCF-7 cells. Among these compounds, lutein appeared to be most cytotoxic, exhibiting approximately 40% inhibition within 24 h of treatment (Fig 4). The rest of the compounds were relatively non-cytotoxic (< 10% cell death). Compared to F3, the individual compounds showed much weaker cytotoxicity indicating that these compounds may not directly contribute to the cell death effect of F3.

## Discussion

Previous studies showed that *S*. *crispus* extracts inhibited the growth of various human cancer cell lines [30–32]. A fraction from the DCM extract was found to be capable of inducing apoptosis of breast and prostate cancer cell lines via the caspase-dependent pathway and displayed relatively higher cytotoxicity than some of the conventional chemotherapeutic agents [11]. However, the *in vivo* cancer therapeutic potential of this plant has never been reported. In the current study, the DCM extract of *S*. *crispus* was chromatographed into five subfractions. The third subfraction (F3) was highly cytotoxic to MCF-7 and MDA-MB-231 breast cancer cells with 80–100% cell death incurred following treatment at the concentration of 100 μg/ml F3. We then further determined the anticancer potential of F3 in NMU-induced breast cancer animal model and analysed the major constituents of this fraction.

Acute oral toxicity studies using crude ethanol extract and juice of *S*. *crispus* leaves, indicated that doses of up to 600 mg/kg [33] and 4,900 mg/kg [34] body weight respectively, were not toxic to female Sprague Dawley rats. In the present study tumor-bearing rats were treated with 40 mg/kg body weight of F3 for eight weeks. This resulted in complete regression of mammary tumors in 3 out of 4 treated animals indicating high efficacy of F3 as an antitumor agent. Although not directly comparable, the same dose of 40 mg/kg body weight was also reported to be optimum for the chemopreventive effect of dietary auraptene in NMU-induced rat mammary tumor model [35]. Nevertheless, further studies are needed in order to establish the optimum antitumor dose of F3. It is also noteworthy that the starting time of treatment differs for each animal due to differences in the tumour incubation periods.

The hematological profile of F3–treated animals showed no signs of anemia, infection or other adverse effects when compared to the normal rats, further supporting the non-toxic nature of F3. The ability of F3 to return the NMU-induced high WBC count towards normal value indicates its potential immunological role in protection against foreign body invasion. On the other hand, significantly low MCH and high platelet counts could also be due to the effect of host factors associated with NMU induction [36, 37]. In agreement with the report of Chang *et al*., [36], we also observed significant loss of body weight with NMU-induced tumor development and treatment with F3 did not improve the body weight in these animals.

Other studies also indicated non-toxicity of *S*. *crispus* extracts. Suherman and colleagues [38] reported no significant effect of *S*. *crispus* water extract on rat liver or plasma microsomal γ-glutamyl transpeptidase (GGT) activities. More recently, a 14-day acute toxicity study using a 5 g/kg body weight daily dose of *S*. *crispus* ethanol extract showed no clinical signs of toxicity in the experimental animals [39]. This was further supported by the study of Lim *et al*, [33] whereby rats repeatedly administered with the ethanol extract of the plant (up to 600 mg/kg body weight) displayed no significant effect on normal liver and kidney functions. *S*. *cripus* extract is also reported to be protective against chemical hepatocarcinogenesis. Supplementation of the extract to Sprague-Dawley rats induced with diethylnitrosamine and 2-acetylaminofluorene showed reduced effect of hepatocarcinogenesis on liver GGT and ALP activities [38] as well as protection against liver cell dysplasia [40].

Membrane-bound (ALP) or cytosolic (ALT & AST) enzymes of liver and kidney cells are used as indices of liver damage in both animals and humans [37]. Variable amounts of these enzymes are leaked into the serum as a result of cellular stress affecting cell membrane integrity [41]. Although the results herein suggest potential development of hepatocellular injury as evidenced by increased serum ALT and AST, and decreased serum ALP levels in F3-treated tumor-bearing rats compared to the normal controls, the changes were not statistically significant. Further studies with longer duration of treatment could confirm any real hepatocellular effects of F3.

Urea, creatinine and uric acid are metabolic by-products of the excess protein, creatine phosphate and purine in the liver and muscle, which are passed into the bloodstream and excreted by kidneys. Low serum concentration of these metabolic waste products could be multifactorial and usually indicates that the kidneys are hyperactive in removing excess fluids and wastes from the blood stream. A similar metabolic state is also obtainable in the presence of impaired liver function [42]. F3 treatment had resulted in some improvement in the renal function indices that were modulated by the tumor induction, particularly the serum creatinine and uric acid levels. As chloride plays a role in maintaining serum acid-base balance, the high level of serum chloride in the F3-treated animals compared to the normal group indicates some impairment of renal function.

Although several *in vitro* and *in vivo* studies in the past had provided some supporting evidences concerning its use against cancer, phytochemical constituents which are responsible for the anticancer effect of *S*. *crispus* remained unclear. The presence of catechins and alkaloids contributes to the high antioxidant capacity of *S*. *crispus* [43] that may offer protection against cancer development via free radical scavenging actions and alleviation of oxidative stress. Stigmasterol and β-sitosterol isolated from *S*. *crispus* leaves were found to be cytotoxic to human cancer cell lines [14]. In the present study, the main chemical constituents of F3 were identified and their potential contribution to the activities of F3 was examined.

Lutein was the predominant compound in F3 as it was isolated in high abundance compared to the other chemical constituents. This compound was also the most active constituent among the other compounds identified in this fraction. Lutein belongs to the xanthophyll family of carotenoids. It differs from other carotenoids in that it has two hydroxyl groups, one on each side of the molecule. These hydroxyl groups were found to play a significant role in the cytotoxic activity of lutein as di-acetylation of the hydroxyl groups resulted in a drop in activity against KB epithelial cancer cells [44]. There is substantial evidence in the literature indicating the correlation between increased dietary intake of lutein with the reduction of risk in contracting cancer owing to its antioxidant, anti-inflammatory and anti-tumour potential activities [45, 46]. Experimental studies in animal models indicated that lutein was able to increase the immunity of mice by enhancing lymphocyte proliferation, resulting in increased tumor latency and suppression of mammary tumor growth at high as well as low dietary levels [47, 48]. The inhibitory effect of lutein on mammary tumor growth was attributed to the ability of the compound to regulate apoptosis and inhibit angiogenesis [49]. Interestingly enough, lutein was also reported to have chemoprotective effect against reactive oxygen species (ROS)-mediated apoptotic cell death of intestinal cells caused by the chemotherapeutic agent, methotrexate [50]. Hence, these indicate that the compound has potential use as an anticancer agent as well as an adjunct to existing chemotherapeutic drugs to prevent oxidative damage caused by the drug towards normal tissues.

Three other chemical constituents found in F3 that bore chlorophyll-like structures were 13^1^-hydroxy-13^2^-oxo-pheophytin a (synonym: purpurin-7-methyl phytyl ester), pheophytin a and 13^2^-hydroxy-pheophytin a (Fig 3). Pheophytin a is a Mg-free analogue of chlorophyll, hence this compound is ubiquitous to all green plants. 13^2^-hydroxy-pheophytin a is an allomerized product of pheophytin a. It has previously been reported in other plants such as *Plagiochila ovalifolia*, *Cordia exaltata* and *Clinacanthus nutans* [29, 51, 52]. Although the other compound, 13^1^-hydroxy-13^2^-oxo-pheophytin a, is expected to exist as an oxidised product of pheophytin a, isolation of this compound from plants has never been reported probably due to its instability and rapid conversion into other allomerized products [53]. Hence, the possibility of isolating this compound in pure and quantifiable form in the present work indicated that the compound is likely to be present in *S*. *crispus* in a reasonably large quantity. Two close derivatives of this compound that have previously been isolated as natural products are purpurin 7-dimethyl ester from *Clerodendrum calamitosum* [54] and purpurin 7-dimethylethyl ester from *Mori folium* [55]. Therefore, 13^1^-hydroxy-13^2^-oxo-pheophytin a seems to be a unique marker for *S*. *crispus*. Interestingly, none of these three compounds were found to be cytotoxic to MCF-7 cells.

Another three chemical constituents found in F3 were campesterol, stigmasterol and β-sitosterol. These sterols that were isolated and tested as a mixture, showed slightly higher cytotoxicity than the pheophytins but are still considered very weak with less than 10% inhibition on the breast cancer cells. Similarly, Lai et al., [56] reported weak cytotoxicity of a mixture of the three phytosterols on HS578T breast cancer cells.

Low cytotoxicity of the individual isolated compounds from F3 suggests that no single chemical constituent identified is solely responsible for the cytotoxic effect of *S*. *crispus* on the breast cancer cell lines. The observed antitumor activity *in vivo* could be attributed to other components of F3 that have yet to be identified. Although the present work has demonstrated the ability of F3 to treat experimental mammary tumor, the therapeutic value of *S*. *crispus* as an anticancer agent may not be limited only to its potent anticancer activities *in vitro* and *in vivo* but also to its ability to synergize with the widely used chemotherapeutic agent, tamoxifen, to induce apoptotic cell death of breast cancer cells, without causing DNA damage to the non-malignant breast epithelial cells [57]. This could potentially reduce the chemotherapeutic dose for treatment of breast cancer with potential reduction in adverse effects. However, potential interactions with other chemotherapeutic agents are currently unknown.

## Conclusion

The work presented herein is pivotal as this is the first report on the therapeutic effect of a bioactive fraction of *S*. *crispus* against mammary tumor *in vivo* and its major chemical constituents. Lutein was the main chemical compound in this fraction, while 13^1^-hydroxy-13^2^-oxo-pheophytin a was isolated for the first time from nature. The other chemical constituents found in the active fraction were campesterol, stigmasterol, β-sitosterol, pheophytin a and 13^2^-hydroxy-pheophytin a. Future work should include delineation of the *in vivo* antitumor mechanism action of F3 and identification of its other components.

## Supporting Information

S1 Fig Completed ARRIVE guidelines checklist. (PDF)

S2 Fig NMR spectra of lutein (1). (PDF)

S3 Fig ESI-MS (+ve) spectrum of lutein (1). (PDF)

S4 Fig NMR spectrum of 131-hydroxy-132-oxo-pheophytin a (2). (PDF)

S5 Fig HR-ESI-MS (+ve) spectrum of 131-hydroxy-132-oxo-pheophytin a (2). (PDF)

S6 Fig NMR spectra of pheophytin a (6). (PDF)

S7 Fig ESI-MS (+ve) spectrum of pheophytin a (6). (PDF)

S8 Fig NMR spectra of 132-hydroxy-pheophytin a (7). (PDF)

S9 Fig MS spectrum of 132-hydroxy-pheophytin a (8). (PDF)
