# Supplementary figures and images for: Anti-Tumor Action, Clinical Biochemistry Profile and Phytochemical Constituents of a Pharmacologically Active Fraction of S. crispus in NMU-Induced Rat Mammary Tumour Model
Source: PLoS One. 2015 May 22;10(5):e0126426. doi: 10.1371/journal.pone.0126426 (PMC4441459; doi:10.1371/journal.pone.0126426)

# $^1\text{H}$ NMR

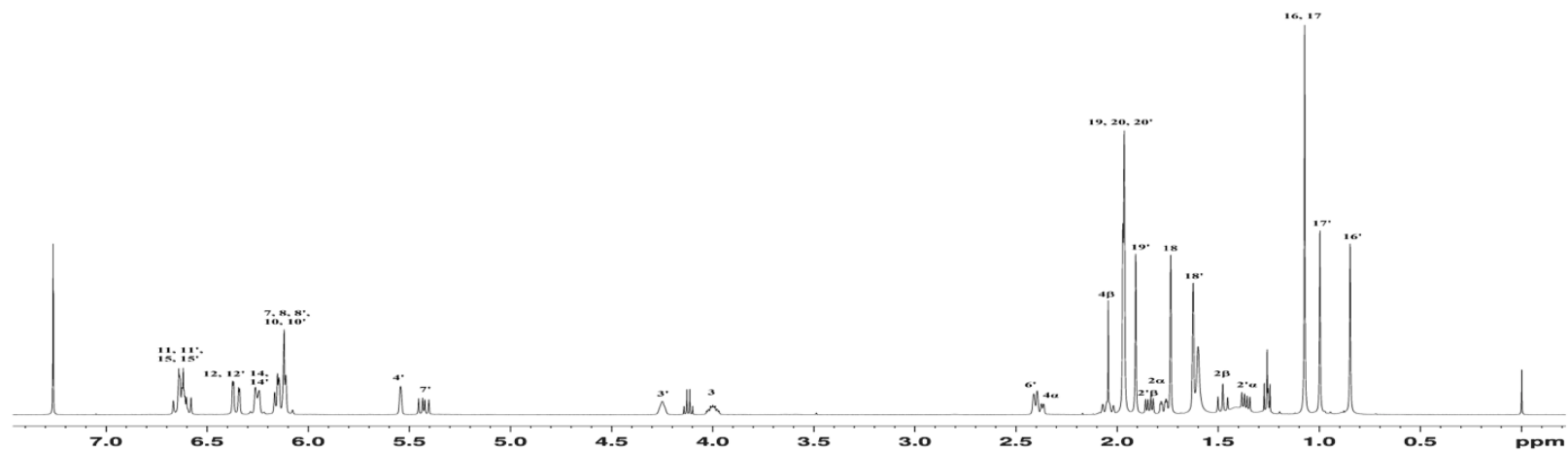

# $^{13}\text{C}$ NMR

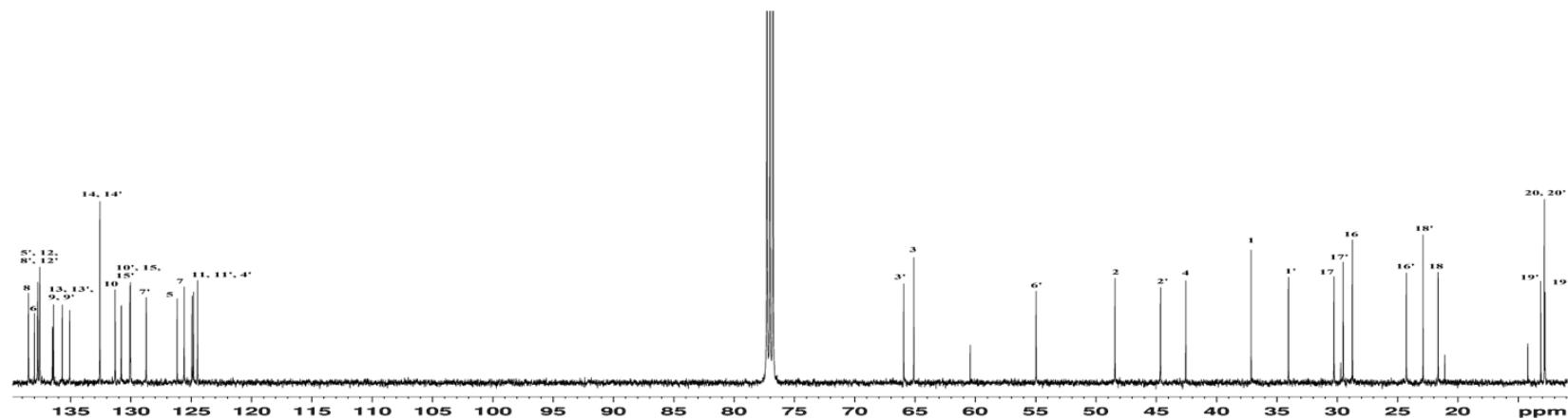

Supplement: S2 Fig — (PDF) [file pone.0126426.s002.pdf]

T: +c ESI Full ms [50.00-1000.00]

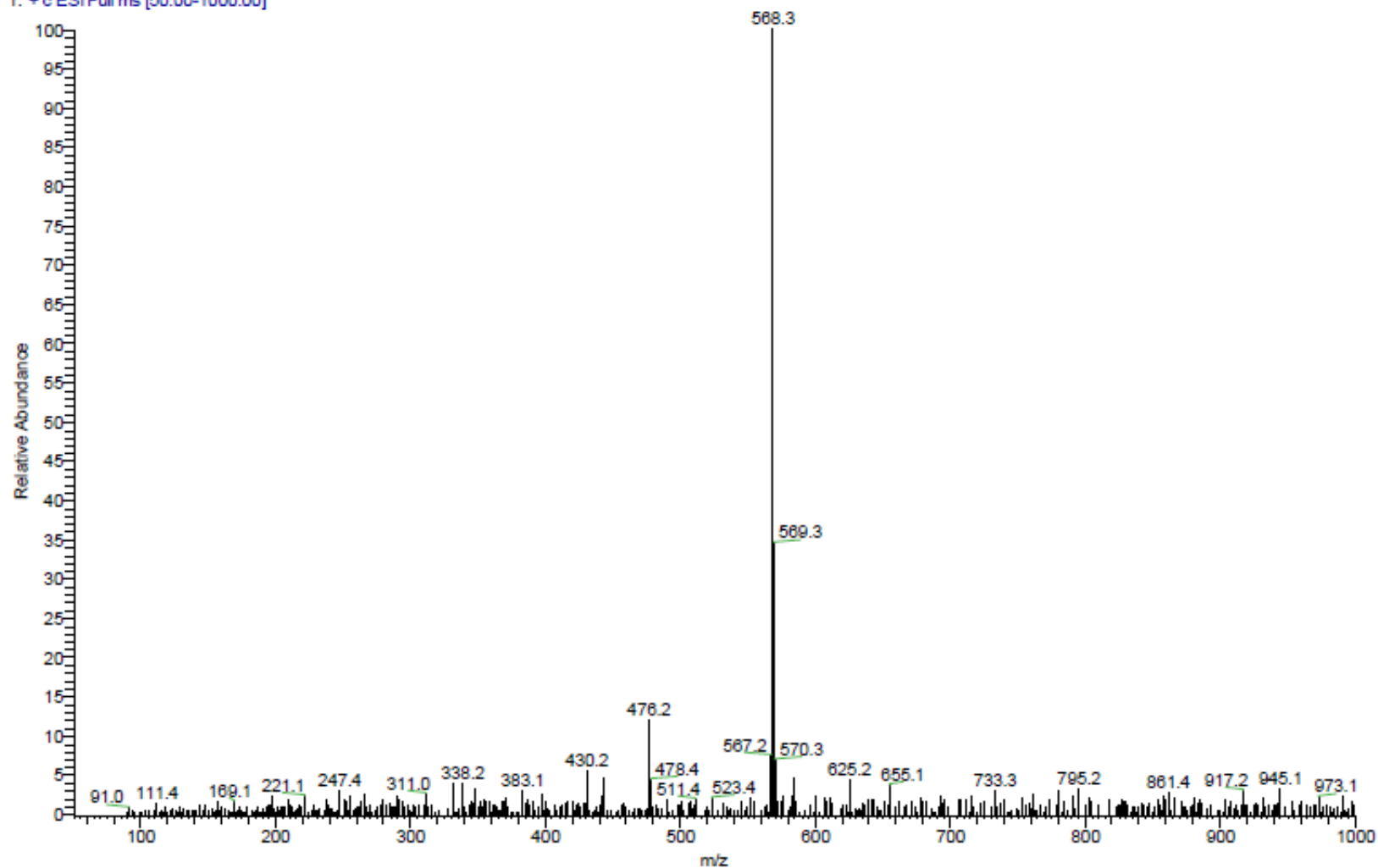

Supplement: S3 Fig — (PDF) [file pone.0126426.s003.pdf]

$^1\text{H}$  NMR

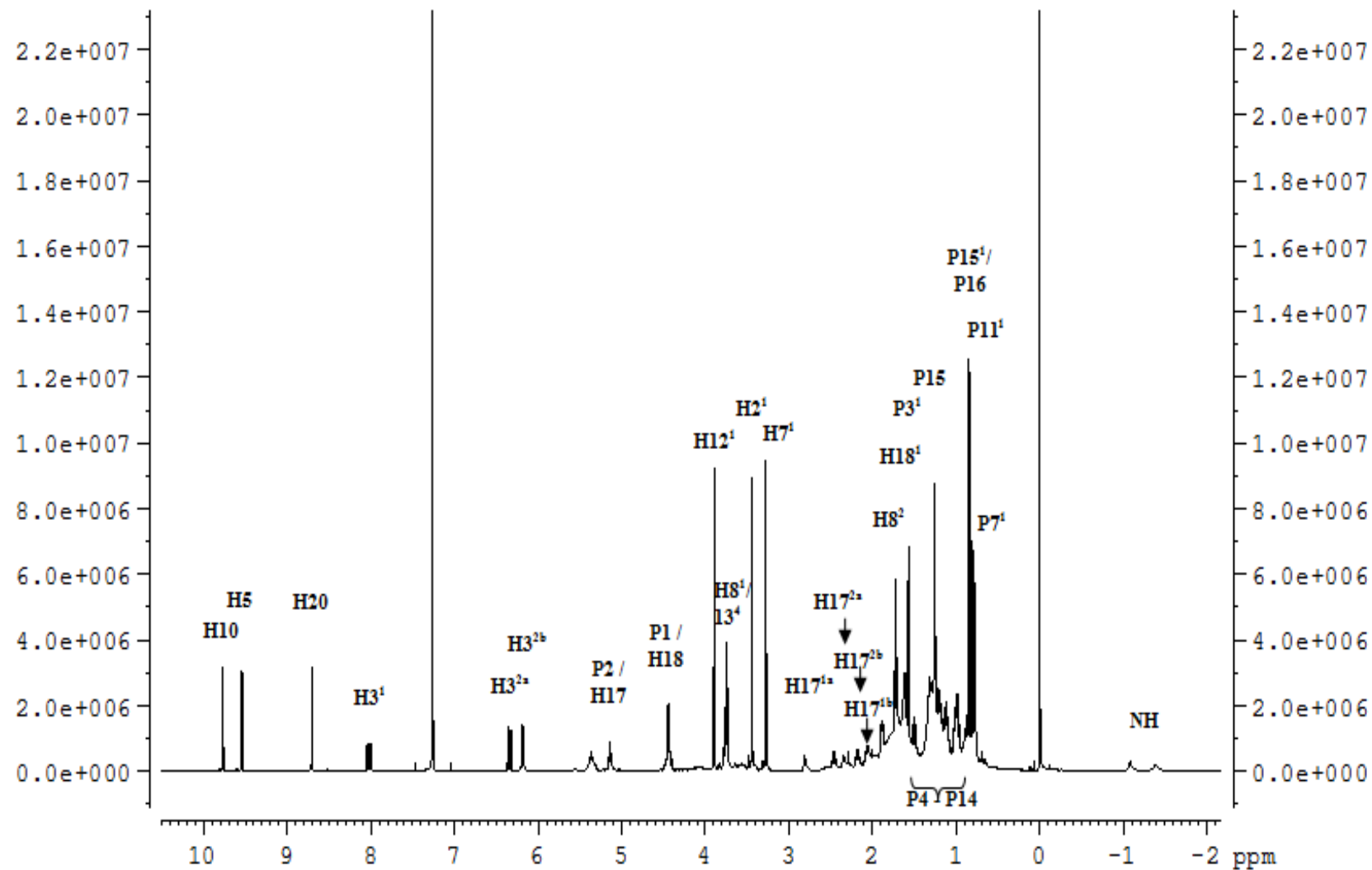

$^{13}\text{C}$  NMR

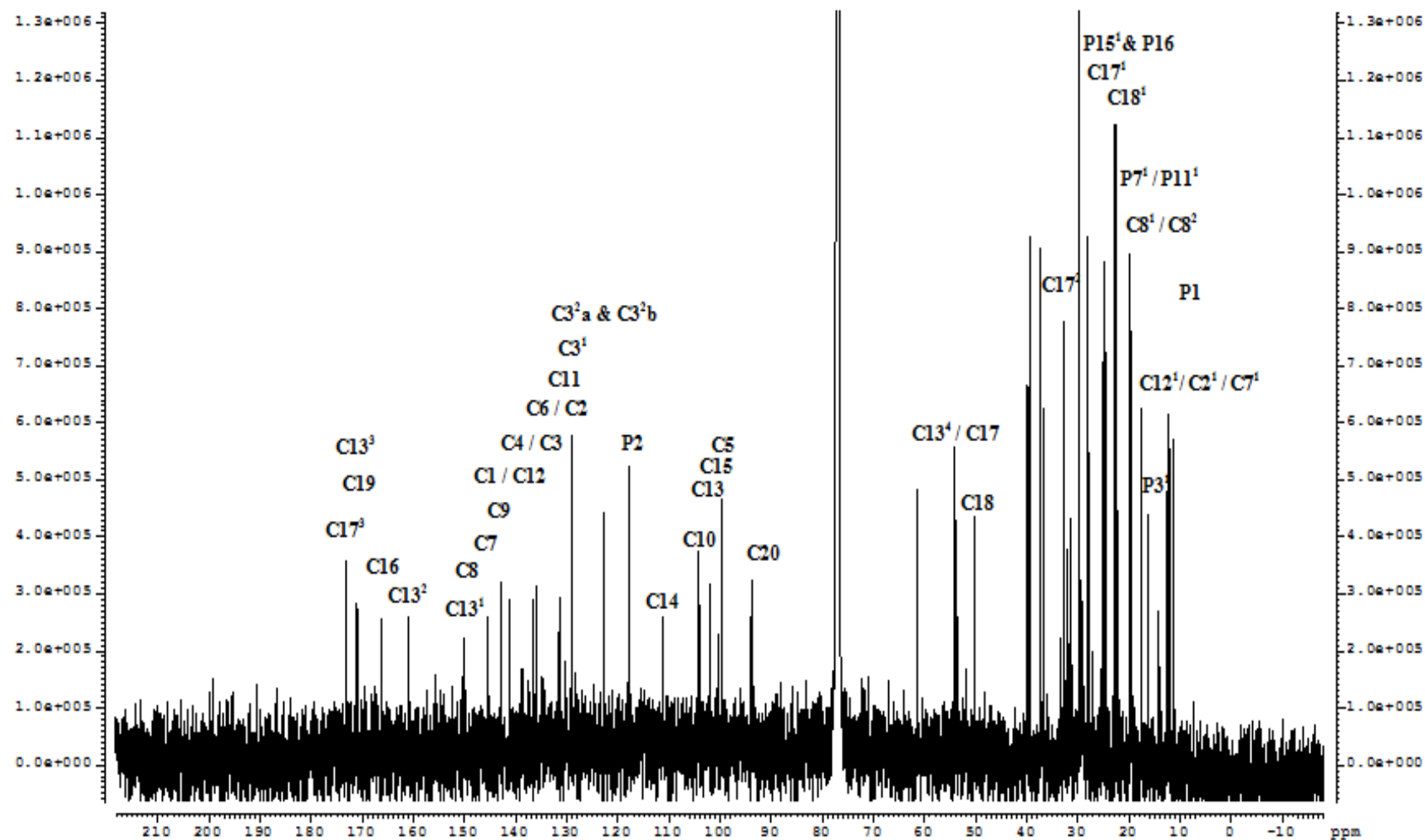

# COSY (2D NMR)

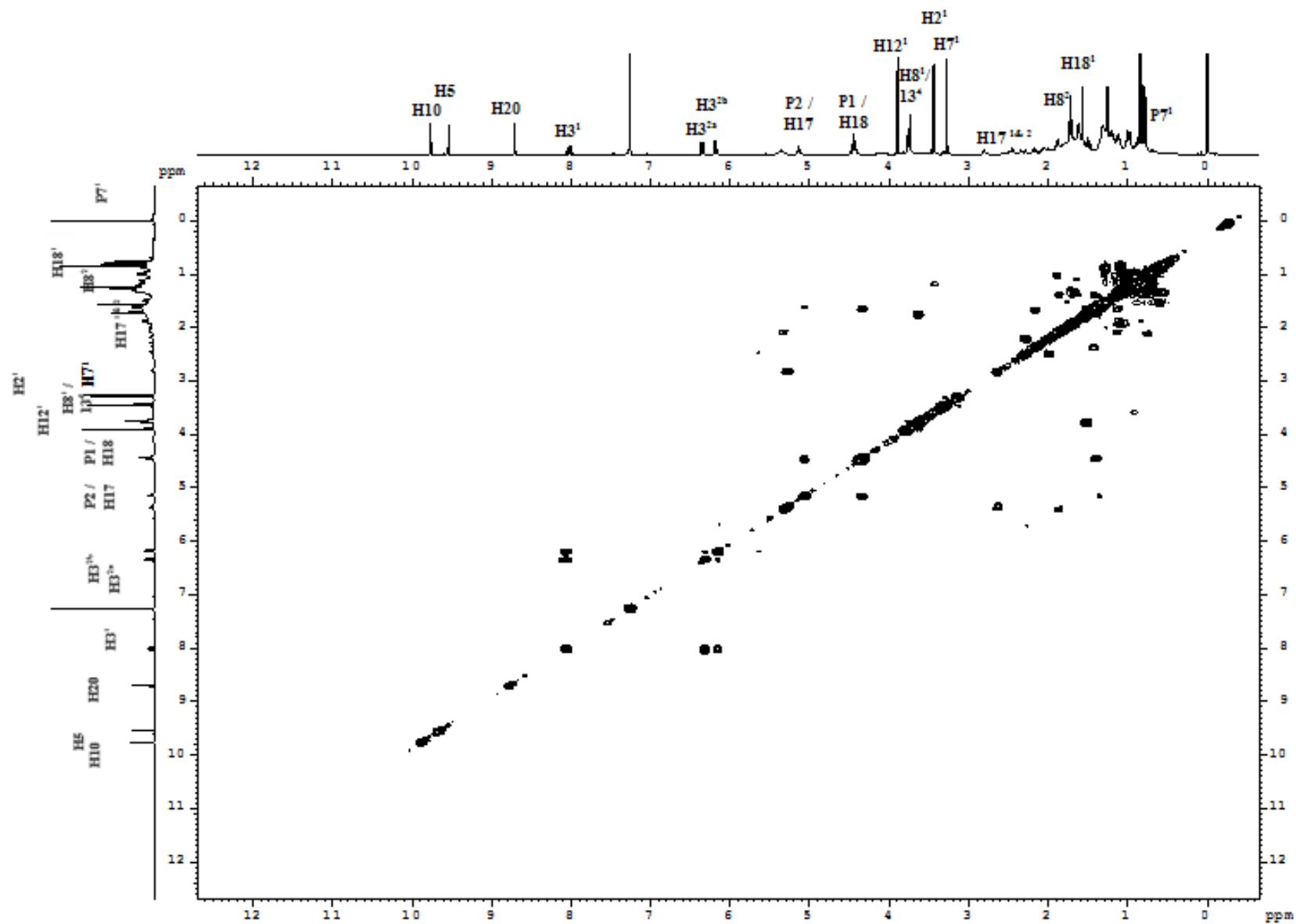

HMBC (2D NMR)

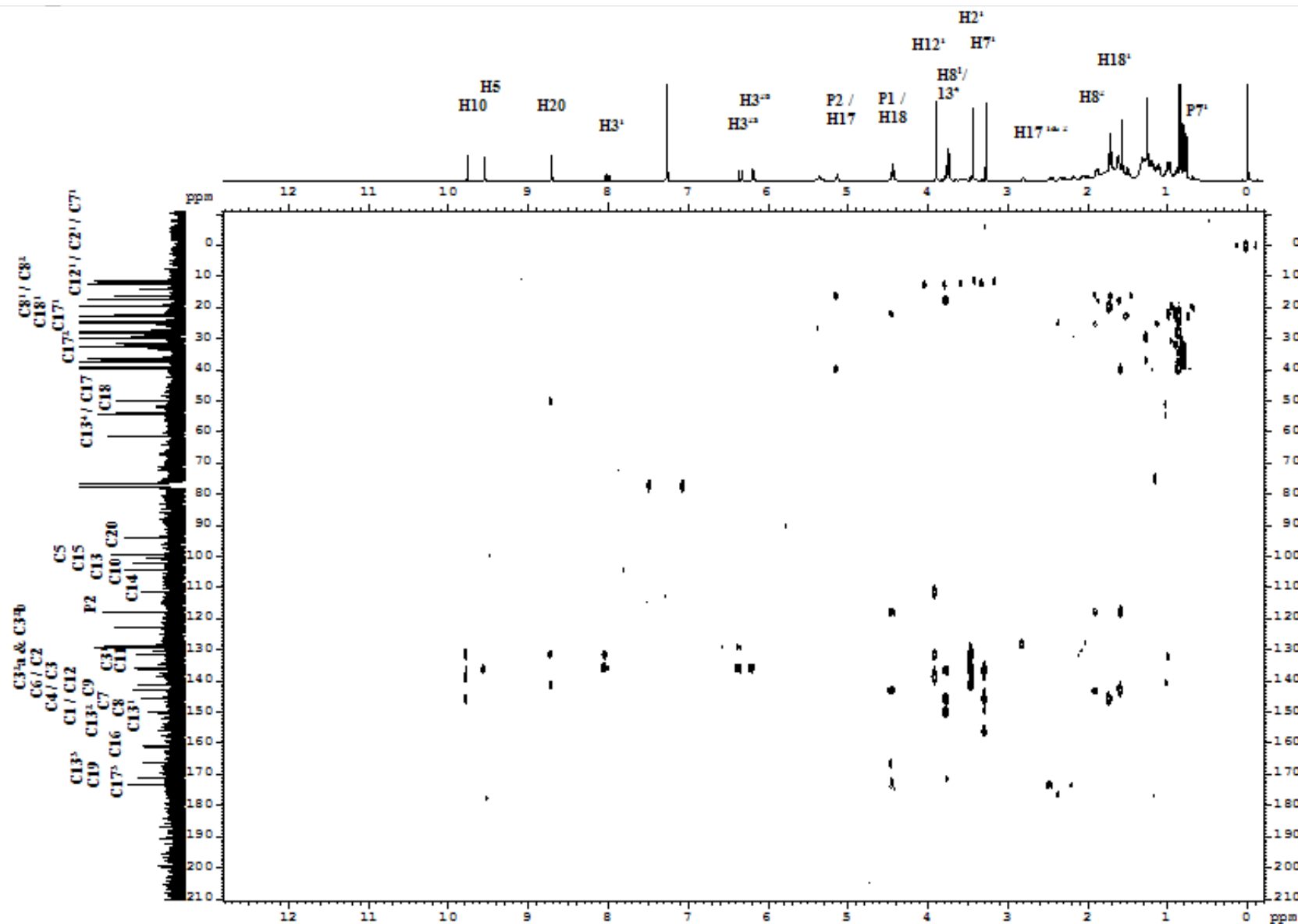

Supplement: S4 Fig — (PDF) [file pone.0126426.s004.pdf]

<sup>1</sup>H NMR

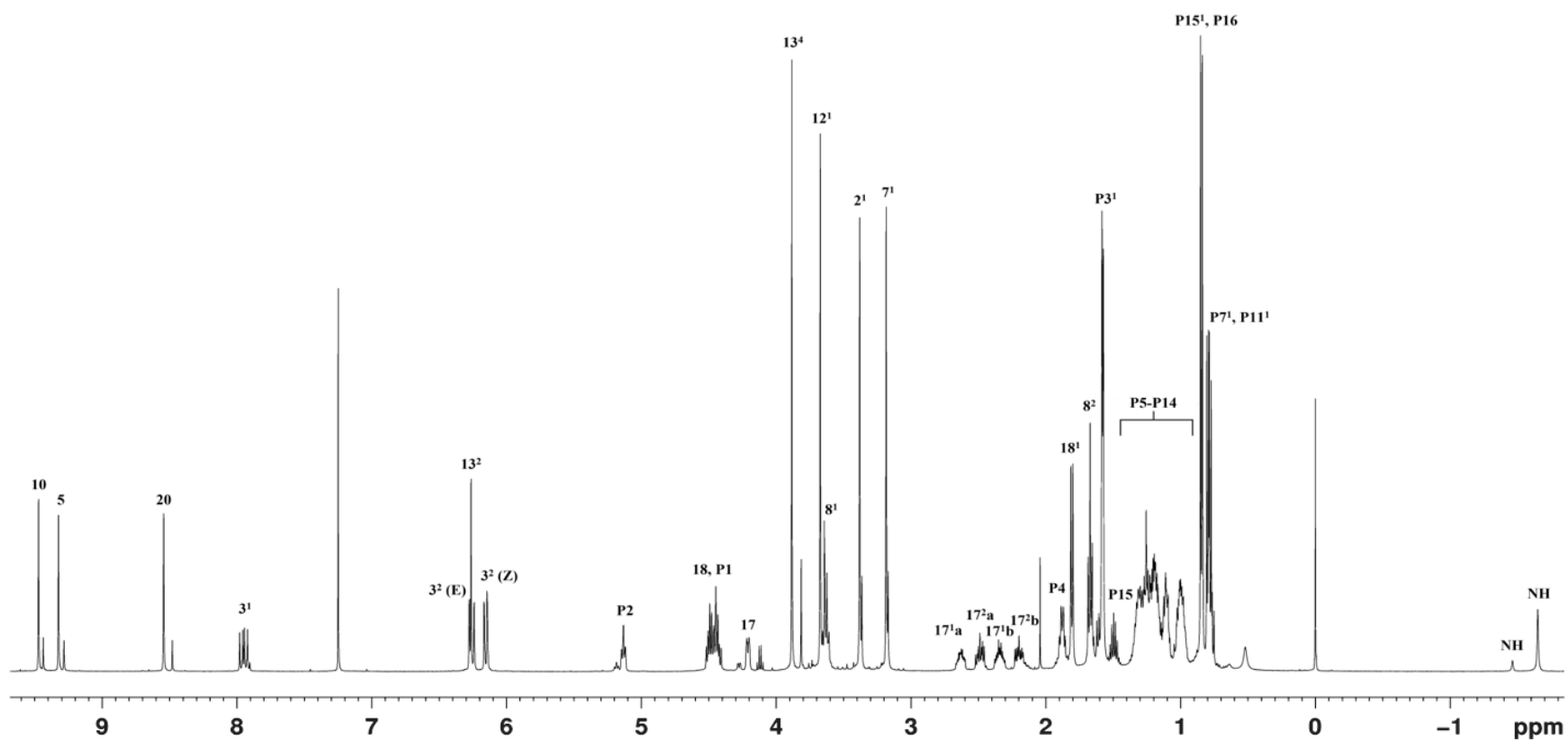

**$^{13}\text{C}$  NMR**

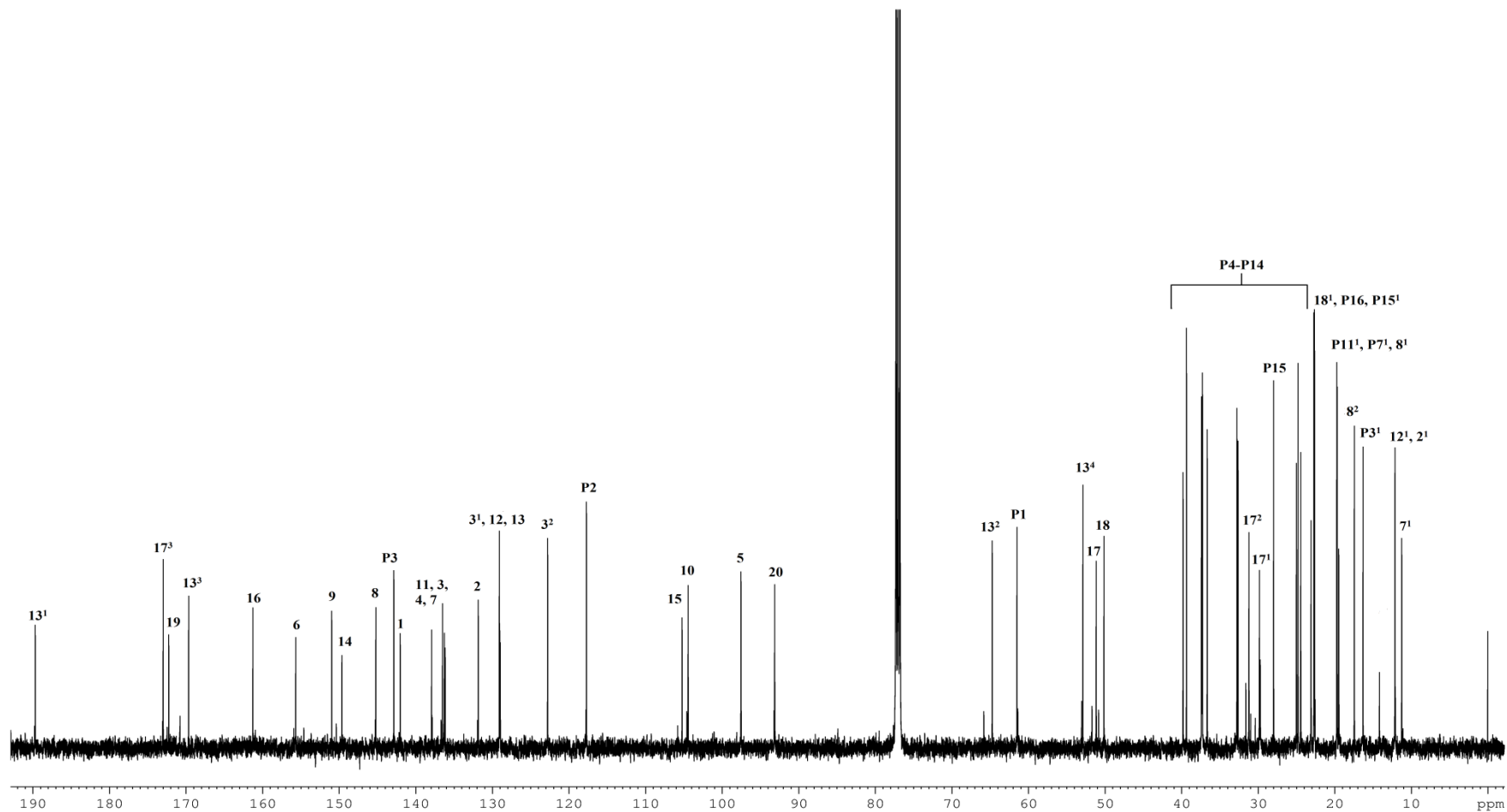

Supplement: S6 Fig — (PDF) [file pone.0126426.s006.pdf]

T: +c ESI Full ms [50.00-1500.00]

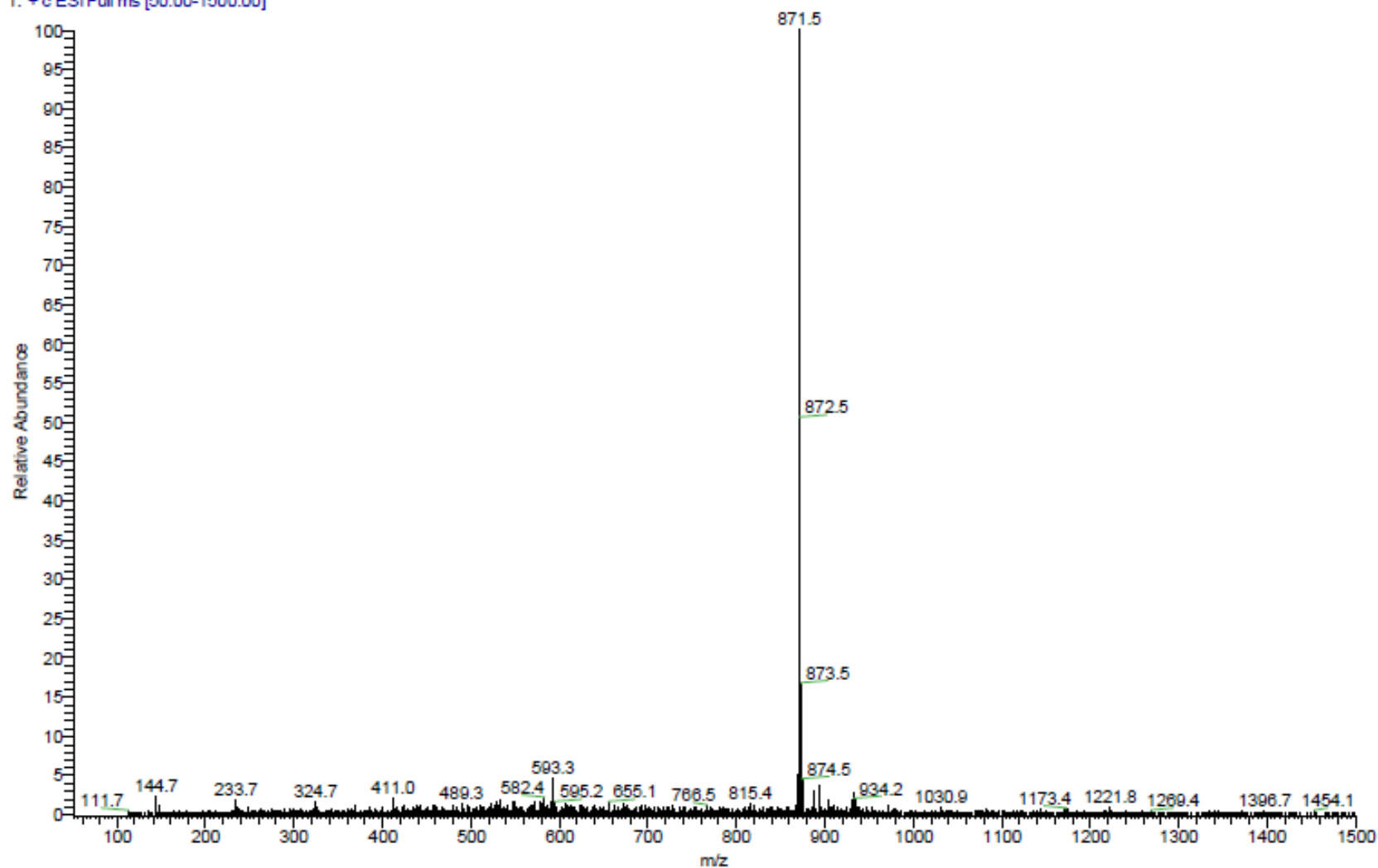

Supplement: S7 Fig — (PDF) [file pone.0126426.s007.pdf]

<sup>1</sup>H NMR

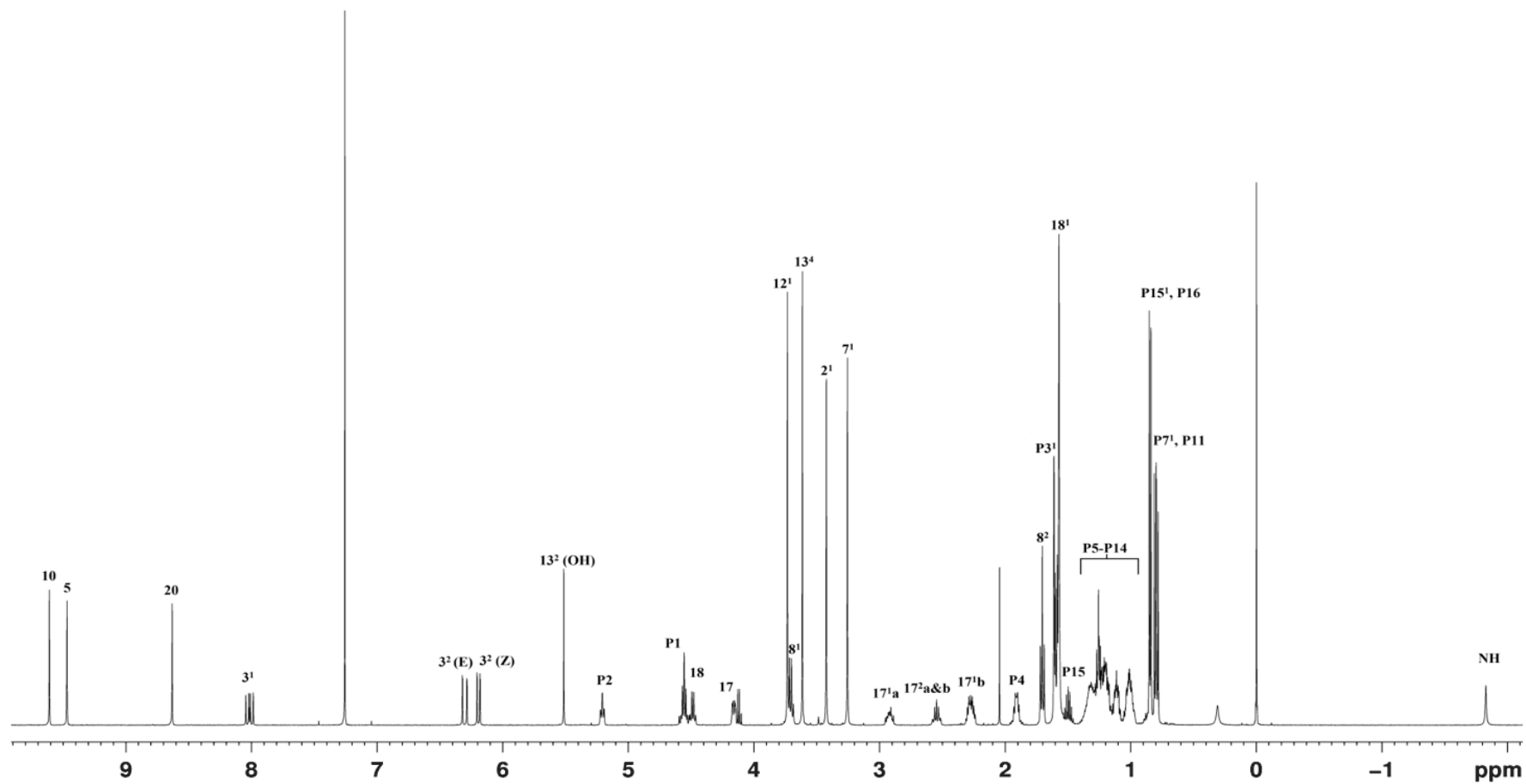

**$^{13}\text{C}$  NMR**

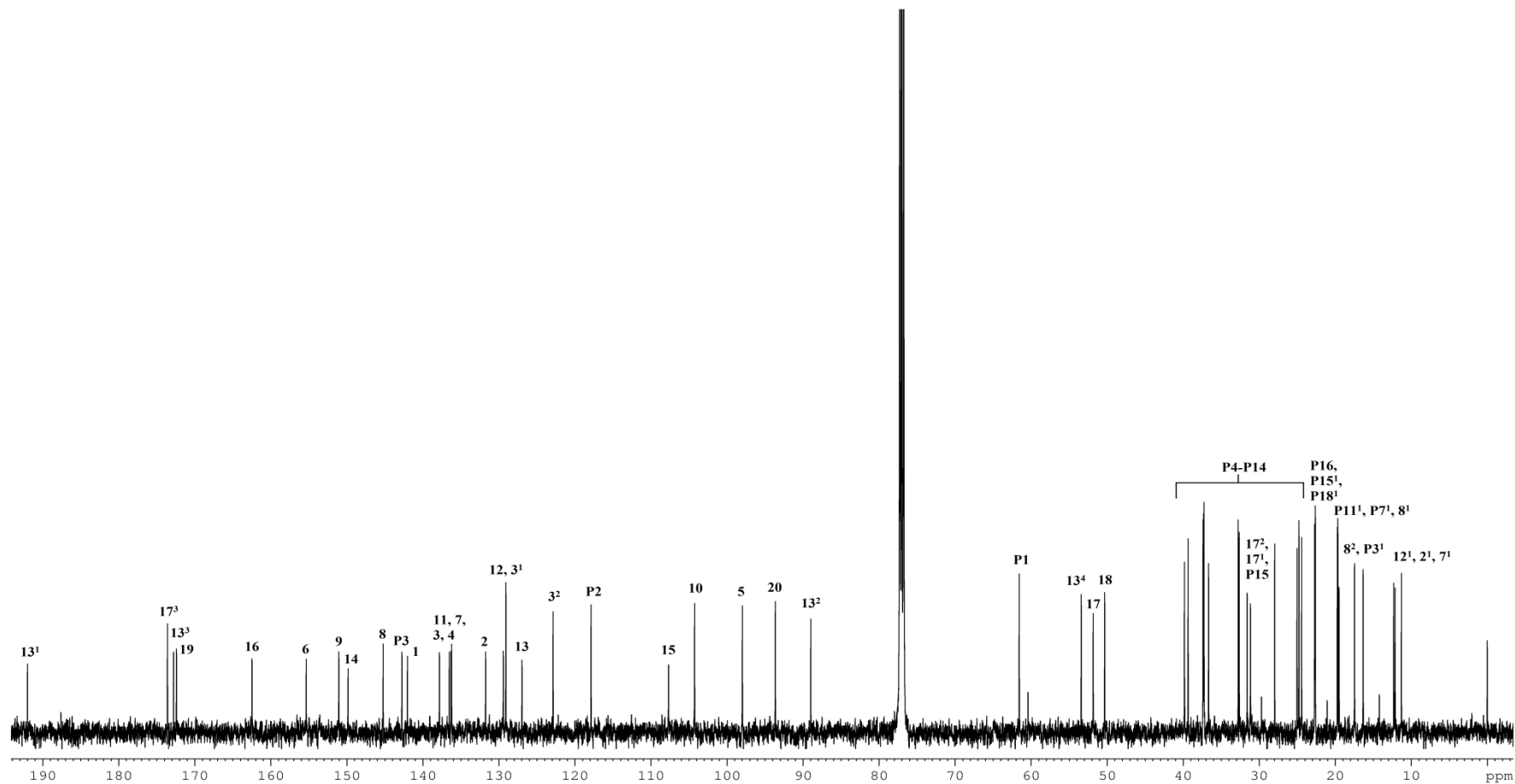

Supplement: S8 Fig — (PDF) [file pone.0126426.s008.pdf]

2014 SC F2 J5 #153-155 RT: 6.01-6.09 AV: 3 SB: 8 4.95-5.20 NL: 1.37E7  
T: +c ESI Full ms [50.00-2000.00]

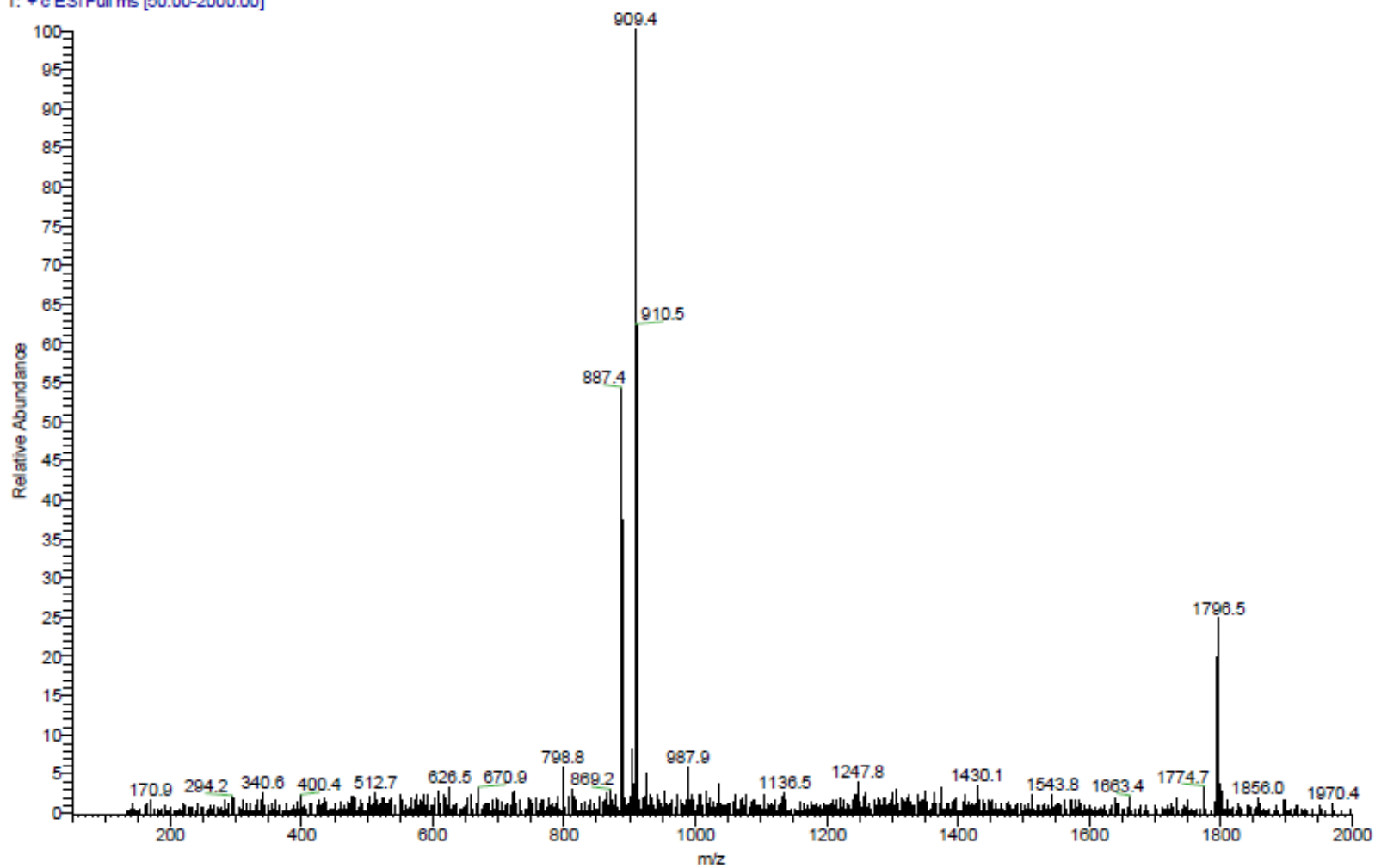

Supplement: S9 Fig — (PDF) [file pone.0126426.s009.pdf]
