# Supplementary material for: Anti-Tumor Action, Clinical Biochemistry Profile and Phytochemical Constituents of a Pharmacologically Active Fraction of S. crispus in NMU-Induced Rat Mammary Tumour Model
Source: PLoS One. 2015 May 22;10(5):e0126426. doi: 10.1371/journal.pone.0126426 (PMC4441459; doi:10.1371/journal.pone.0126426)

Meas. m/z	#	Formula	m/z	err [ppm]	rdb	e⁻ Conf	N-Rule
903.5657	1	C 58 H 76 N 2 Na O 5	903.5646	-1.1	21.5	even	ok
	2	C 60 H 75 N 2 O 5	903.5670	1.5	24.5	even	ok
	3	C 58 H 73 N 5 O 4	903.5657	0.1	25.0	odd	ok
	4	C 55 H 75 N 4 O 7	903.5630	-2.9	20.5	even	ok
	5	C 56 H 74 N 5 Na O 4	903.5633	-2.6	22.0	odd	ok

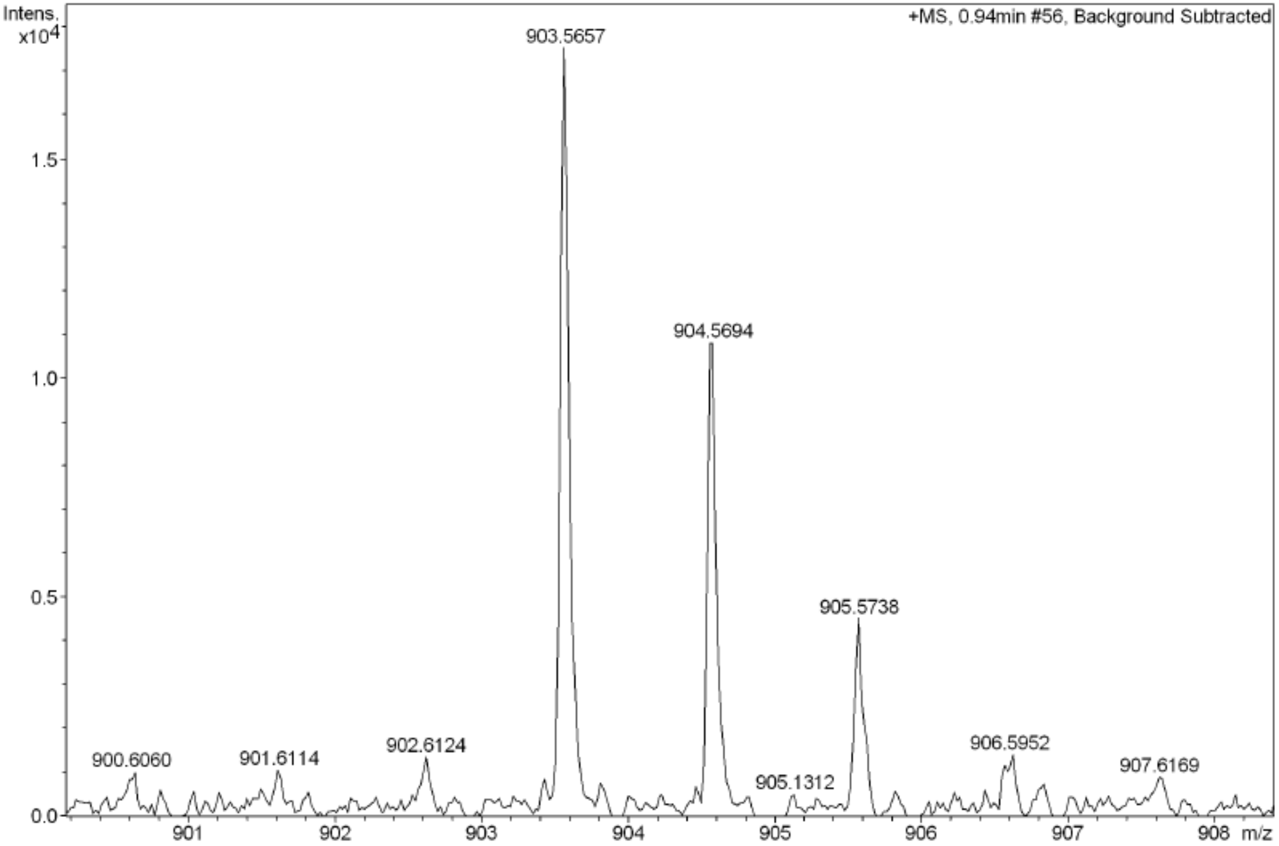

Supplement: S5 Fig — (PDF) [file pone.0126426.s005.pdf]
